# Land tenure contributions to protected area growth under alternative conservation targets in the Australian monsoon tropics

**DOI:** 10.1111/cobi.70143

**Published:** 2025-09-09

**Authors:** Emmeline Norris, Ben Scheele, Marcel Cardillo

**Affiliations:** ^1^ Research School of Biology Australian National University Canberra Australian Capital Territory Australia; ^2^ Centre for Tropical Environmental and Sustainability Science, College of Science and Engineering James Cook University Cairns Queensland Australia; ^3^ Fenner School of Environment and Society Australian National University Canberra Australian Capital Territory Australia

**Keywords:** extinction, Indigenous land, mammals, pastoral land, prioritization, systematic conservation planning, threatened species, especies amenazadas, extinción, mamíferos, planeación sistemática de la conservación, priorización, tierras indígenas, tierras de pastoreo, 灭绝, 土著土地, 哺乳动物, 牧场, 优先保护, 系统性保护规划, 受威胁物种

## Abstract

As the global protected area (PA) network expands to meet international targets, it is important to assess whether traditional reliance on public land will suffice for projected PA growth or whether other tenures, such as Indigenous or pastoral lands, may increasingly contribute. Another consideration is whether the relative importance of different tenures varies depending on the specific goals of the PA network. We used the mammal fauna of the Australian monsoon tropics (AMT), one of the world's largest intact tropical savannas, as a case study to address these questions. We applied systematic conservation planning to identify optimal PA configurations under 2 objectives (adding to the existing PA network from any tenure vs. expanding the Indigenous protected area [IPA] network through voluntary declaration of Indigenous lands by traditional owners) and 2 species protection criteria (prioritizing currently threatened species vs. species predicted to become threatened). We calculated planning unit selection frequencies for the resulting 4 scenarios to identify high‐priority areas for mammal conservation and assessed their dependence on different tenure categories. All scenarios relied heavily on Indigenous lands to achieve species representation targets, with varying contributions from pastoral land depending on the criteria prioritized. Protecting potentially threatened species required more pastoral land and Indigenous land coexisting with primary industries, whereas targets for currently threatened species were more cost‐effectively met through voluntary declarations of Indigenous freehold land as IPAs. Our results highlight the potential for Indigenous lands to play a major role in achieving biodiversity conservation targets and demonstrate that land tenure requirements vary depending on conservation priorities. These findings emphasize the need to explicitly consider tenure in conservation planning to guide collaborative strategies and ensure PA growth aligns with specific biodiversity goals across diverse land management contexts.

## Abstract (es)

Contribuciones de la tenencia de la tierra al crecimiento de las áreas protegidas bajo objetivos alternativos de conservación en los trópicos australianos monzónicos

**Resumen:**

A medida que la red mundial de áreas protegidas (AP) se expande para cumplir con los objetivos internacionales, es importante evaluar si la dependencia tradicional de las tierras públicas será suficiente para el crecimiento previsto de las AP o si otras formas de tenencia, como las tierras indígenas o de pastoreo, pueden contribuir cada vez más. Otra consideración es si la importancia relativa de las diferentes formas de tenencia varía en función de los objetivos específicos de la red de AP. Utilizamos los mamíferos de los trópicos monzónicos australianos (TMA), una de las sabanas tropicales intactas más extensas del mundo, como estudio de caso para abordar estas cuestiones. Aplicamos una planificación sistemática de la conservación para identificar las configuraciones óptimas de áreas protegidas en función de dos objetivos (añadir a la red de áreas protegidas existente desde cualquier régimen de tenencia frente a ampliar la red de áreas protegidas indígenas (API) mediante la declaración voluntaria de tierras indígenas por parte de los propietarios tradicionales) y dos criterios de protección de especies (dar prioridad a las especies amenazadas actualmente frente a las especies que se prevé que se vean amenazadas). Calculamos las frecuencias de selección de unidades de planificación para los cuatro escenarios resultantes con el fin de identificar las áreas de alta prioridad para la conservación de mamíferos y evaluamos su dependencia de las diferentes categorías de tenencia. Todos los escenarios dependían en gran medida de las tierras indígenas para alcanzar los objetivos de representación de especies, con contribuciones variables de las tierras de pastoreo en función de los criterios priorizados. La protección de especies potencialmente amenazadas requería más tierras de pastoreo y tierras indígenas que coexistieran con las industrias primarias, mientras que los objetivos para las especies actualmente amenazadas se cumplían de manera más rentable mediante declaraciones voluntarias de tierras indígenas en propiedad como API. Nuestros resultados resaltan el potencial de las tierras indígenas para desempeñar un papel importante en la obtención de los objetivos de conservación de la biodiversidad y demuestran que los requisitos de tenencia de la tierra varían en función de las prioridades de conservación. Estas conclusiones subrayan la necesidad de considerar explícitamente la tenencia en la planificación de la conservación para orientar las estrategias de colaboración y garantizar que el crecimiento de las áreas protegidas se ajuste a los objetivos específicos de biodiversidad en diversos contextos de gestión de la tierra.

## 【摘要】

全球保护地网络正在持续扩张以实现国际目标, 因此, 评估依赖公共土地的传统模式能否满足保护地扩张的预期需求, 或是土著土地、牧场等其他所有制类型的土地是否产生越来越多的帮助, 具有重要意义。此外, 另一个关键问题在于, 不同所有制类型土地的相对重要性是否随保护地网络的具体目标而变化？本研究以澳大利亚季风热带区(全球最大的原始热带稀树草原之一)哺乳动物群落作为案例, 探讨了上述问题。我们应用系统性保护规划方法, 在两种目标(利用所有土地类型扩大现有保护地网络, 或通过传统土地所有者自愿划定土著土地来扩大土著保护地网络)及两种物种保护标准(优先保护现存受威胁物种, 或优先保护预测将受威胁物种)下, 确定了保护地的最佳规划方案。我们计算了四种情景下规划管理单元的选择频率, 以确定哺乳动物保护的高优先区域, 并评估其对不同土地类型的依赖性。我们发现, 所有情景均高度依赖土著土地以实现物种代表性目标, 而牧场土地的贡献程度则因优先保护标准而异。保护潜在受威胁物种需要更多牧场土地与土著土地来共同支持初级产业, 而通过土著将自由保有的土地自愿划定为土著保护地, 则可以更具成本效益地实现现存受威胁物种的保护。以上研究结果强调了土著土地在实现生物多样性保护目标中的潜在重要作用, 并表明对土地所有权类型的要求因优先保护规划而异。这些发现强调了应在保护规划中明确考虑土地所有权问题, 以指导合作策略, 确保保护地扩张符合不同土地管理方式下的具体生物多样性目标。**【翻译:胡怡思;审校:聂永刚】**

## INTRODUCTION

Globally, biodiversity conservation relies heavily on government‐owned and government‐managed protected areas (PAs) (Bingham, Lewis, et al., 2021; UNEP‐WCMC & IUCN, 2024). Areas outside PA networks often have high biodiversity value, and there is growing recognition that planning for effective conservation across large regions must include diverse forms of tenure, including Indigenous‐managed lands (Bingham, Fitzsimons, et al., 2021; Fitzsimons & Wescott, 2008; O'Bryan et al., 2021; Schuster et al., 2019). For example, in Australia, Brazil, and Canada, Indigenous lands support vertebrate diversity equivalent to PAs and exceeding unprotected areas (Schuster et al., 2019), and in Mexico, Indigenous‐managed conservation areas have greater mammal diversity than government‐managed PAs (Padilla et al., 2025). At a global scale, Indigenous lands provide habitat for 60% of all mammal species (O'Bryan et al., 2021). This may reflect lower human population densities, traditional land stewardship practices (Yibarbuk et al., 2001), and increasing recognition of Indigenous land rights that enable restrictions on extractive industries to prioritize conservation management (Blackman et al., 2017; Ceddia et al., 2015; Holden, 2005). Indigenous peoples own or manage approximately 38 million km^2^ of land worldwide (Garnett et al., 2018), positioning these lands as potentially important contributors to achieving the Convention on Biological Diversity's (CBD) goal of protecting 30% of the world's land by 2030 (30×30 goal) set in the Kunming–Montreal Global Biodiversity Framework (GBF) (CBD, 2022). The GBF strongly emphasizes integrating traditional knowledge with evidence‐based science, recognizing the rights and roles of Indigenous communities in global biodiversity conservation (Newing et al., 2024).

In Australia, Indigenous and pastoral lands are important parts of the conservation management landscape. Many private and government‐managed PAs occur on pastoral leasehold or Native Title land, which does not grant exclusive rights to Indigenous people and may coexist with primary industries, such as grazing (Fitzsimons, 2015). Indigenous protected areas (IPAs) are areas under Indigenous ownership that have been declared as part of Australia's National Reserve System (NRS) following voluntary agreement by Indigenous traditional owners and managed for the maintenance of biocultural diversity (DCCEEW, 2024; NIAA, 2015). In recent years, the total area of IPAs has grown more rapidly than PAs under public or private ownership, now comprising over half of Australia's NRS (Farr et al., 2016). Although livestock grazing on native vegetation is the dominant land use in Australia, accounting for half of Australian land (Thackway, 2018), the total area used for grazing has decreased in the past decade, largely due to government acquisition of pastoral leases for conversion to PAs (Williams et al., 2021).

As in many countries, additions to Australia's NRS by declaration of IPAs and acquisition of pastoral leases tend to happen opportunistically. Consequently, the PA network in Australia has generally grown in an ad hoc fashion (Adams et al., 2011). Currently, around 22% of Australia's total land area is incorporated in PAs (Fitzsimons et al., 2023), meaning an additional 600,000 km^2^ of land must be protected to achieve the CBD's 30×30 goal, to which Australia has committed (Commonwealth of Australia, 2019). With limited funding for conservation, there is a strong imperative to plan more strategically for the continuing expansion of the NRS. This can be achieved by applying principles of systematic conservation planning (SCP): defining a priori conservation goals at the national or regional scale and then identifying land additions that maximize goal attainment while minimizing PA establishment and management costs (Bonebrake et al., 2019; Hoffmann, 2022; Kukkala & Moilanen, 2013; Margules & Pressey, 2000).

To effectively implement SCP principles, 2 key questions must be addressed. First, what should the goals of a PA network be? When it comes to prioritizing species, the prevailing approach emphasizes the protection of currently threatened or endemic species, which are assumed to be most at risk of extinction and, hence, the most urgent priorities for protection (Farrier et al., 2007; Kraus et al., 2023). In Australia, this is made explicit in the Commonwealth Government's overarching policy statement on biodiversity conservation (DCCEEW, 2022b). However, there are increasing calls for species‐based conservation to become more proactive in outlook by identifying and protecting species that may not yet be threatened but are considered likely to become threatened in the future (Cardillo et al., 2006, 2023; Lunney et al., 2004; Peng et al., 2023). It remains unclear whether different conservation goals that emphasize current versus anticipated future threats to biodiversity might lead to different conservation planning priorities.

Second, what is the relative importance of land tenures and management regimes in achieving conservation goals? This is an important question because areas under different tenures vary not only in their biodiversity values but in the costs or impediments associated with PA establishment and ongoing management (Miteva et al., 2019). For example, acquisition costs associated with establishing IPAs are likely to be lower than for converting pastoral leases because traditional owners can voluntarily declare IPAs, whereas governments must usually purchase pastoral leases at market value (Adams et al., 2011). Pastoral lease conversions may also incur higher opportunity costs due to foregone agricultural production; however, environmental degradation associated with overgrazing is also estimated to incur significant opportunity costs, which could be offset by implementing sustainable land management practices (Russell‐Smith & Sangha, 2018). Similarly, costs associated with converting private freehold land depend on whether landholders enter voluntary Nature Refuge covenants, or the land is purchased by the state (Adams et al., 2011). In contrast, allocating Crown land to the NRS involves no acquisition costs (Fitzsimons et al., 2023).

As a case study to investigate these questions, we used the native mammal fauna of the Australian monsoon tropics (AMT), a large, biodiverse region with a relatively even balance of public PAs, Indigenous lands, and pastoral leases. The AMT (Appendix S1) covers 1.3 million km^2^ in northern Australia and includes extensive tropical savanna ecosystems, sandstone escarpments, laterite plateaux, riparian forests, and wetlands. Only a small proportion has been converted to intensive agricultural landscapes. The area under pastoral leasehold (20% of the AMT) is used mostly for cattle grazing in partially intact natural vegetation systems. Around 45% of the AMT is under Native Title. Aboriginal Land Councils or Corporations hold 60% of Native Title land under inalienable Indigenous freehold title. Indigenous land‐use agreements (ILUAs) exist on a further 38% of non‐Native Title land and recognize traditional owners’ rights to access and use public lands managed for industries, such as mining and pastoralism, without requiring Native Title determination (Fitzsimons et al., 2023; NNTT, 2021).

Approximately 178 mammal species (60% of Australia's extant native mammal fauna) inhabit the AMT. The Kimberley, Top End, and Cape York regions are hotspots of species richness and endemism (Appendix S1). Many mammal species’ populations across northern Australia have declined severely in recent decades (Woinarski et al., 2011; Ziembicki et al., 2015). The primary threats to mammals in the AMT are thought to be introduced species, in particular cats (*Felis catus*) and, in the southern, drier parts of the region, foxes (*Vulpes vulpes*) (Fairfax, 2019; Murphy et al., 2019; Woinarski et al., 2011). Cane toads (*Rhinella marina*) have spread east to west across the AMT over the past 40 years (Doody et al., 2018; Phillips et al., 2007) and are likely responsible for population declines in native predators, such as the northern quoll (*Dasyurus hallucatus*) (Webb et al., 2015). In part of the pastoral zone, the introduction of highly combustible African pasture grasses, including buffelgrass (*Cenchrus ciliaris*) and gamba grass (*Andropogon gayanus*), may also have degraded habitat quality for mammals by shifting the seasonal cycle of small, low‐intensity understory fires toward more destructive high‐intensity wildfires in the late dry season (Martin et al., 2015; Setterfield et al., 2010).

Using the AMT mammal fauna as a case study, we aimed to determine how conservation planning outcomes differ under biodiversity goals that prioritize the protection of currently threatened species versus species predicted to become threatened. Specifically, we examined the extent to which achieving representation targets under these goals depends on different land tenures, particularly Indigenous and pastoral lands. To address these aims, we used comparative models of extinction risk to quantify mammal species’ inherent sensitivity to human impacts as a basis for inferring the potential for currently unthreatened species to become threatened. We then constructed alternative species representation targets based on potential future threat status and applied area selection algorithms to identify priority areas for addition to the existing PA network in the AMT. Finally, we assessed how optimal PA configurations under future risk targets differ in land tenure dependencies compared with the prevailing approach for expanding the PA network, which heavily emphasizes currently threatened species. By applying an SCP framework that explicitly considers land tenure, we sought to identify strategies for optimizing PA expansion to meet global and local conservation goals and to highlight opportunities for early engagement and cross‐cultural collaboration.

## METHODS

### Spatial, phylogenetic, and biological data

We used the Interim Biogeographic Regionalization for Australia (IBRA) 7.0 framework (DCCEEW, 2021) to delineate our study area, the AMT monsoon phytogeographical subregion (Brundrett, 2017) (Appendix S1). This subregion is composed of 18 IBRA bioregions. We excluded the adjacent Wet Tropics bioregion because it is predominantly composed of closed‐canopy rainforests rarely affected by fire and has a unique, highly endemic faunal composition. We used a 1 × 1‐km grid overlaid on the AMT as the base for all spatial operations. We used R 4.3.1 statistical software for all analyses (R Core Team, 2023).

We obtained geographic distributions of AMT mammal species as spatial polygons from the International Union for Conservation of Nature (IUCN) (IUCN, 2023). We downloaded occurrence records for the non‐native red fox and cane toad from the Atlas of Living Australia (ALA, 2023a, 2023b) and removed duplicate records, records lacking coordinates, and records with coordinate uncertainty >2 km. We then converted each of these to a binary variable indicating presence or absence in each grid cell. Feral cats were excluded from the models because their widespread presence in Australia (Legge et al., 2017) prevents meaningful analysis of overlap with native mammals based on occurrence data.

As biological predictors in the comparative models, we obtained species‐level summary values of adult body mass, age at first reproduction, number of litters per year, and litter size from the COMBINE data set (Soria et al., 2021) for AMT mammal species to be included in the comparative extinction risk models. These variables reflect key life‐history variation in mammal species that underlies population density and growth rates and are known predictors of mammal threat status (Cardillo et al., 2008, 2006; González‐Suárez & Revilla, 2013).

We used data on 2 climate variables (mean temperature of the wettest quarter and precipitation of the driest quarter) from WorldClim (Fick & Hijmans, 2017) to capture the interactions between growth and accumulation of invasive C4 grasses in the wet season and the susceptibility of the landscape to intense fires in the dry season. To explore the direct effect of fire on total fire frequency and late dry season fire frequency, we used spatial data from Northern Australia and Rangelands Fire Information (NAFI, 2021).

As an indicator of anthropogenic land modification, we used the human influence index (HII), which captures human population density, distance to transport infrastructure, light pollution, land use, and built‐up areas, to classify the degree of human impact on a 0‐ to 61‐point scale (Sanderson et al., 2002; WCS & CIESIN, 2005). For use in comparative models, we summarized each spatial variable as a mean across the distribution of each mammal species.

To serve as the phylogenetic framework for comparative models, we took a random sample of 100 phylogenies from a Bayesian posterior set of mammal phylogenies (Upham et al., 2019) and pruned these to include Australian native species only.

### Comparative models and spatial patterns of extinction risk

We fitted comparative models of extinction risk for 128 native terrestrial mammal species found in the AMT for which the data set was complete. The final suite of mammal species was obtained by removing 2 data‐deficient species and 14 species listed under IUCN criterion B (small geographic range size) to reduce circularity in the models (Cardillo et al., 2008). We excluded extinct species from the models due to insufficient data on biological traits (e.g., body mass) or geographic distribution. We removed an additional 16 species with unresolved phylogenetic placement and 18 species with insufficient (<2%) geographic overlap and hence spatial data for the AMT (e.g., species that predominantly occur in the Pilbara and Central Desert regions).

We transformed predictor variables to minimize skewness of residuals and then standardized to units of standard deviation. As the response variable for the models, we used the IUCN categories assigned to the final subset of AMT mammals (IUCN, 2024). We combined the 5 categories (least concern, near threatened, vulnerable, endangered, critically endangered) with associated estimates of recent population trend (increasing, stable, decreasing, unknown) to produce a 10‐point ordinal variable (Appendix S5).

To fit models, we used a generalized least squares (GLS) regression that accounts for possible phylogenetic and spatial nonindependence in the data (Bromham et al., 2022). We derived phylogenetic covariances from the global mammal phylogenies and used Euclidean distances between centroids of species distributions as spatial covariances. We used variance inflation factor (VIF) to assess covariates for collinearity and removed the precipitation of the driest quarter variable due to its strong negative correlation with both total fire frequency and temperature of the wettest quarter.

As an initial step, we fitted a full model with the 11 predictor variables plus a quadratic term for body mass to account for expected increased extinction risk in medium‐sized mammals (Johnson & Isaac, 2009). We then used a heuristic stepwise approach to find a minimum adequate model (MAM) by successively deleting the predictor with the highest *p* value and comparing the new model's performance with its successor, considering ΔAICc ≥2 as the criterion for an improved model fit, until all remaining predictors were important additions to the model. We then sequentially added each deleted predictor back into the model until we found a new MAM. We used diagnostic checks on the MAM to ensure assumptions of normality and homogeneity of residuals were met and fitted the final MAM with 100 randomly selected phylogenies to account for additional variance introduced by phylogenetic uncertainty.

For each of the 100 models, we calculated latent extinction risk for each species as its fitted value from the model minus its current threat status (Cardillo et al., 2006), as coded on our 10‐point numeric scale. For further analyses, we used the mean of latent risk values across the 100 models, for each species.

To visualize the spatial distribution of extinction risk, we mapped the total and mean values of current and positive latent risk. Total risk is the sum of values for all species in each grid cell, so it captures the mean level of extinction risk and species richness. To aggregate latent risk across species, we used the mean of positive (>0) latent risk values because it is the positive values that suggest the potential for future risk increases and that are therefore of greatest interest for proactive conservation planning.

### Systematic conservation planning

To identify priority areas for expanding the PA network across the AMT, we used the R package prioritizr (Hanson et al., 2022, 2024), which uses exact integer linear programming to find optimal conservation solutions based on a customizable algorithm. The algorithm requires as input a spatial planning unit (PU) layer containing data on PU costs and values of the biodiversity features to be optimized. The user can then customize the conservation problem by setting constraints (e.g., areas that must be included or excluded in the solution) and the relative protection targets for each conservation feature. Due to the AMT's vast area (∼1.3 million km^2^), the existing 1 × 1‐km grid, comprising 1,346,466 cells across the AMT, imposed computational limitations preventing its use as the PU layer in prioritization scenarios. Hence, we created a tessellation of 10,064 hexagonal PUs with side lengths of 7.5 km, perimeter of 45 km, and maximum area of 146 km^2^ and summarized spatial data to these polygons. Some PUs were cropped to the AMT boundary and therefore smaller in area. To adjust for PU size variation in the prioritizr algorithm, we weighted proxy costs and feature data by each PU's proportion of a full‐sized PU.

We classified all PUs as being a PA; IPA (DCCEEW, 2022a); Indigenous freehold; Native Title land; ILUA (NNTT, 2021); pastoral term or perpetual lease; or other freehold, term, perpetual lease, or Crown lands (ABARES, 2021). Each PU was classified to whichever form of tenure accounted for more than 50% of its area. Where Native Title land or ILUAs overlapped with the pastoral lease classification, these PUs were classified as Native Title land pastoral use and ILUA pastoral use, respectively (Appendix S3).

To create a layer that represented the costs of establishing and managing PAs, we considered proxies for land value. Land value data are scarce in Australia (Chancellor et al., 2019); broadacre land prices were only available for the state of Queensland (Business Queensland, 2022). In Queensland, the spatial distribution of mean land value approximates that of median employee income (MEI) 2011–2018 census data (ABS, 2018); therefore, across the AMT jurisdictions (Queensland, Northern Territory, and Western Australia), we used MEI as a proxy for land value (Appendix S2).

To account for increased land values in proximity to infrastructure and urban centers (Chancellor et al., 2019), we combined MEI with HII by extracting the modal MEI and HII values for PUs, normalizing these values to between 0 and 1 and scaling the resultant values to between 0.01 and 100 to avoid overrepresentation of low‐cost PUs (Gurobi Optimization, 2022). We then weighted the MEI and HII values by 0.4 and 0.6, respectively, to attribute greater importance to the well‐established influence of urban development on land value in the final proxy cost values: cost=0.4MEI+0.6HII.

The final proxy cost layer thus comprises measures of local economic productivity and urbanization to support the cost‐effective selection of PUs in the AMT (Appendix S2). We did not factor land tenure into the proxy cost calculation to avoid biasing the prioritization algorithm toward certain tenures.

To optimize PU selection for addition to the existing PA network, we specified 2 criteria for determining species representation targets. The first criterion, species’ current threat status according to the IUCN Red List, was based on current conservation priorities. The second criterion was based on positive values of latent risk, which indicated species that are currently less threatened than predicted by the comparative models and that may therefore have greater potential to become threatened (Cardillo et al., 2006).

Representation targets (i.e., the proportion of a species’ distribution included in prioritizr solutions) were generated for the 10% of species with the highest extinction risk values under the respective criterion. This equated to all 15 AMT mammal species classified as endangered or critically endangered for the current threat status criterion and 13 species with a latent risk value >1.50 for the positive latent risk criterion. All other species were given zero targets to reflect the trend of higher resource allocation to protect species considered high priorities. We calculated representation targets as the weighted sum of the target species’ threat status and the proportion of their geographic distribution in the AMT.

The proportion of species’ AMT distributions to be protected (P) under the current and latent risk criteria was specified as P=0.4R+0.6A, where A is the proportion of the species’ distribution in the AMT and R is the value of current or latent risk on the pseudo‐numeric scale, scaled between 0.25 and 0.75 to avoid extremely high or low representation targets. We limited representation targets to a maximum of 75% of a species range to ensure all targets could feasibly be met by the prioritization algorithm (Appendix S6).

For each representation criterion, we defined 2 conservation objectives aimed at meeting the representation targets: expanding the current PA network by adding to it from any form of land tenure (i.e., Indigenous, public, private, or Crown lands) (Objective 1) and expanding the IPA network through voluntary declaration of Native Title and Indigenous freehold land by traditional owners (Objective 2).

The fundamental aims for all criteria and objectives were to meet species representation targets for the minimum possible cost; to find a single optimal solution for each scenario; and, in Objective 2, to exclude PUs overlaying tenure other than Native Title or Indigenous freehold land. For each optimal solution, we calculated the total area, boundary length, and proxy cost; the overall representation of AMT mammal species; and the proportion of selected PUs in each land tenure category.

Finally, we calculated the selection frequency of PUs across each of the 4 scenarios (i.e., combination of criteria and objectives). This provided a way to summarize the relative importance of PUs across a range of scenarios.

Derived data sets and R code to reproduce the results of this study can be downloaded from https://zenodo.org/records/15142190 and https://zenodo.org/records/13821433, respectively (Norris, 2024, 2025).

## RESULTS

### Comparative models and spatial patterns of extinction risk

The MAM for 128 mammals in the AMT explained 32.80% of the variation in current threat status (Table 1). The MAM (AICc = 480.11) was chosen over the full model (AICc = 486.82) and included indicators of life‐history speed (body mass, age at first reproduction, litters per year), geographic range size, distributional overlap with foxes and cane toads, and total and late dry season fire frequency (Table 1; Appendix S4). Latent risk values ranged from strongly negative (–4.89 for the spectacled flying‐fox [*Pteropus conspicillatus*]), indicating species that were already more threatened than the model predicted, to strongly positive (2.59 for the Arnhem sheath‐tailed bat [*Taphozous kapalgensis*]), indicating species currently less threatened than the model predicts (Appendix S5).

**TABLE 1:** Summaries of the full and minimum adequate models of extinction risk produced using generalized least squares regression and a single phylogeny for 128 terrestrial mammal species native to the Australian monsoon tropics.

	Full model (AICc = 486.82)^a^, ^b^				Reduced model (AICc = 480.11)^b^			
Predictor	Slope estimate	SE	*t*	*p*	Slope estimate	SE	*t*	*p*
Intercept	2.421	0.209	11.581	0.0000^***^	2.401	0.205	11.700	0.0000^***^
Life history								
Adult body mass	0.562	0.174	3.233	0.0016^**^	0.562	0.171	3.286	0.0013^***^
(Adult body mass)^2^	−0.276	0.166	−1.660	0.0997^*^	−0.250	0.160	−1.560	0.1214^#^
Age of first reproduction	0.611	0.266	2.296	0.0235^*^	0.669	0.237	2.802	0.0059^**^
Litters per year	0.342	0.241	1.421	0.1581	0.358	0.233	1.534	0.1276
Litter size	−0.114	0.196	−0.582	0.5616				
Ecological								
Geographic range size	−0.714	0.277	−2.573	0.0114^*^	−0.630	0.244	−2.588	0.0109^*^
Range overlap with foxes	0.557	0.279	2.003	0.0475^*^	0.531	0.273	1.942	0.0545^*^
Range overlap with cane toads	−0.308	0.195	−1.582	0.1164^#^	−0.283	0.169	−1.681	0.0955^#^
Environmental								
Temperature of wettest quarter	−0.044	0.264	−0.165	0.8692				
Human influence	−0.075	0.222	−0.337	0.7368				
Total fire frequency	0.906	0.263	3.444	0.0008^***^	0.947	0.232	4.084	0.0001^***^
Late dry season fire frequency	−0.738	0.222	−3.329	0.0012^**^	−0.741	0.200	−3.699	0.0003^***^

^a^ Akaike's information criterion values corrected for small sample size (AICc) indicate that the outputs of the full and minimum adequate models differ significantly from each other (ΔAICc = 6.71), and the minimum adequate model provides a better model fit.

^b^ Significance: ^#^ *p* ≤ 0.5; *p ≤ 0.1; **p ≤ 0.01; ***p ≤ 0.001.

**FIGURE 1:**
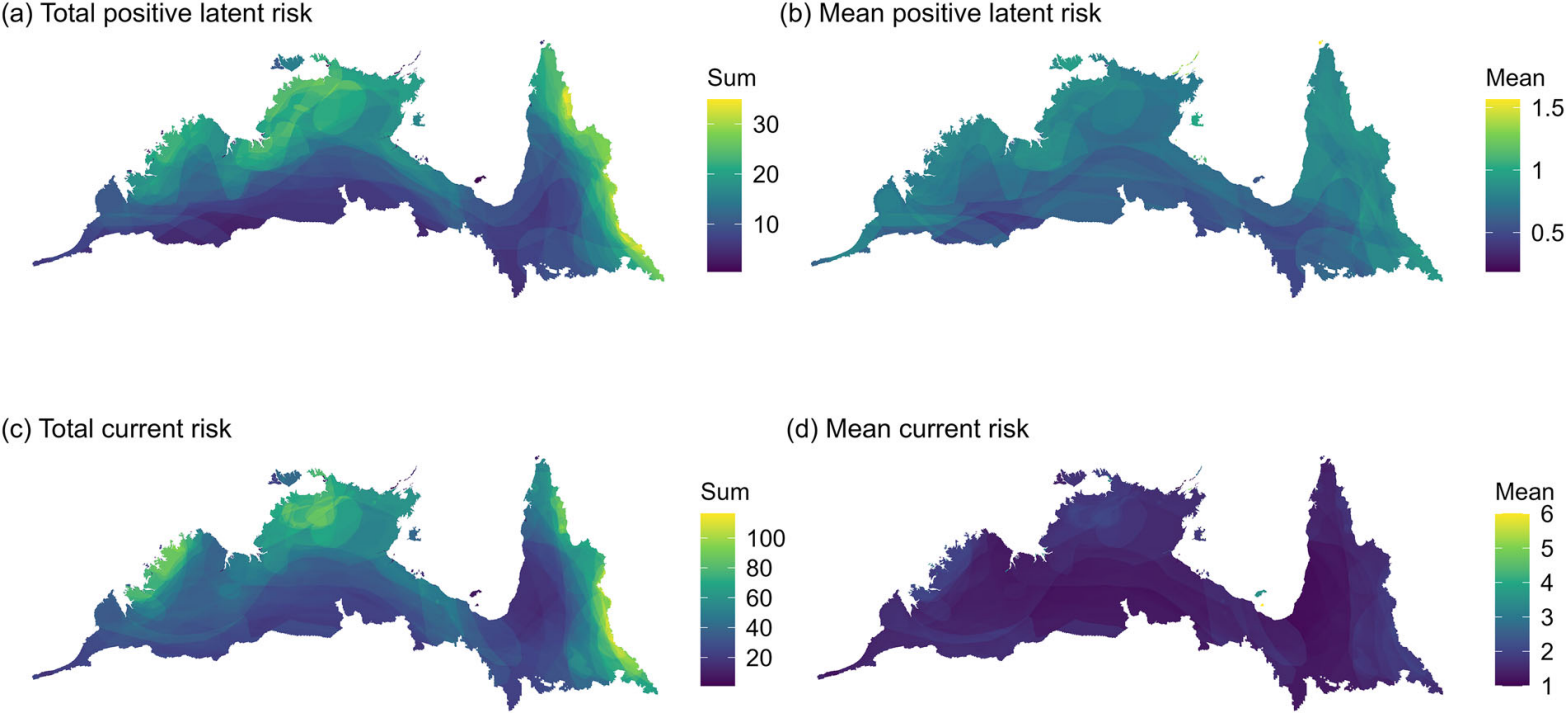
In the Australian monsoon tropics, patterns of (a) total positive latent, (b) mean positive latent, (c) total current, and (d) mean current risk of extinction for 128 native terrestrial mammal species calculated for 1‐km^2^ raster grid cells. The wet tropics bioregion was excluded from analyses.

There was considerable heterogeneity in risk across the AMT (Figure 1). Total current and positive latent risk closely reflected patterns of overall species richness and were highest in the Einasleigh Uplands, the Darwin Coastal, and Arnhem Plateau bioregions of the Northern Territory and in the Northern Kimberley in Western Australia (Figure 1a,c; Appendix S1). Hotspots of total positive latent risk were in Kutini–Payamu (Iron Range) National Park in Cape York Peninsula and the Victoria Bonaparte bioregion abutting the Bonaparte Gap (Figure 1a; Appendix S1). Mean positive latent risk was highest in the Thursday Island cluster, Wessel Islands, Pellew Islands, and Maria Island (Figure 1b), and mean current risk was highest in the Wellesley Islands and Inglis Island off the Northern Territory coast (Figure 1d), reflecting the disproportionately high extinction risk of island endemic mammal species because these islands have lower species richness than nearby areas on mainland Australia (Appendix S7).

### Systematic conservation planning

**FIGURE 2:**
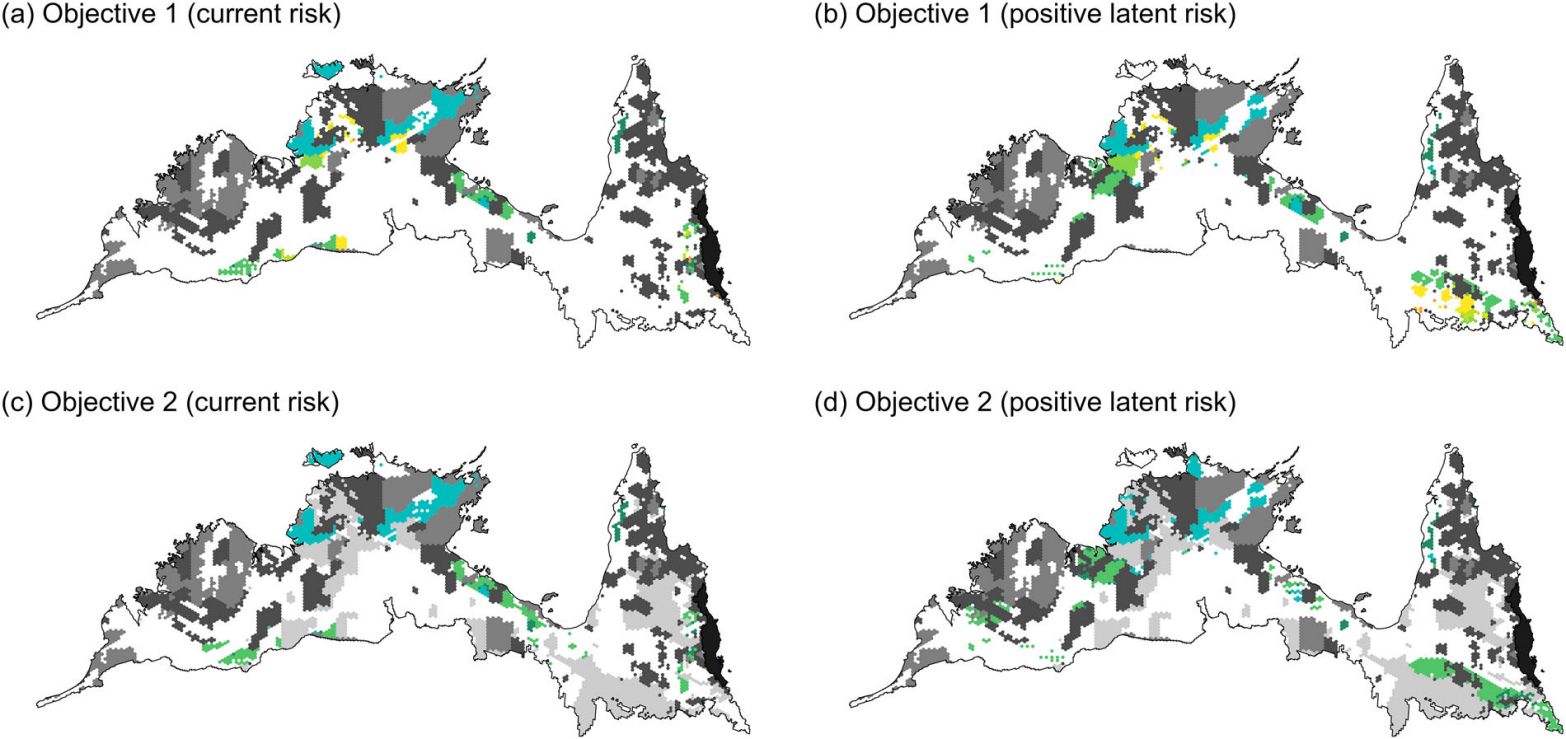
Optimal protected area (PA) configurations for expansion of PAs by adding to the existing reserve network from any tenure (Objective 1) and increasing the Indigenous protected area (IPA) network through voluntary declarations of Native title and Indigenous freehold land by traditional owners (Objective 2) when prioritizing current risk versus positive latent risk of extinction of mammal species native to the Australian monsoon tropics (light gray shading in [c] and [d], areas not under Native Title or Indigenous freehold and not included in selection for second objective; black shading, Wet Tropics bioregion excluded from analyses; white, areas available for selection but not selected in optimal solutions).

**FIGURE 3:**
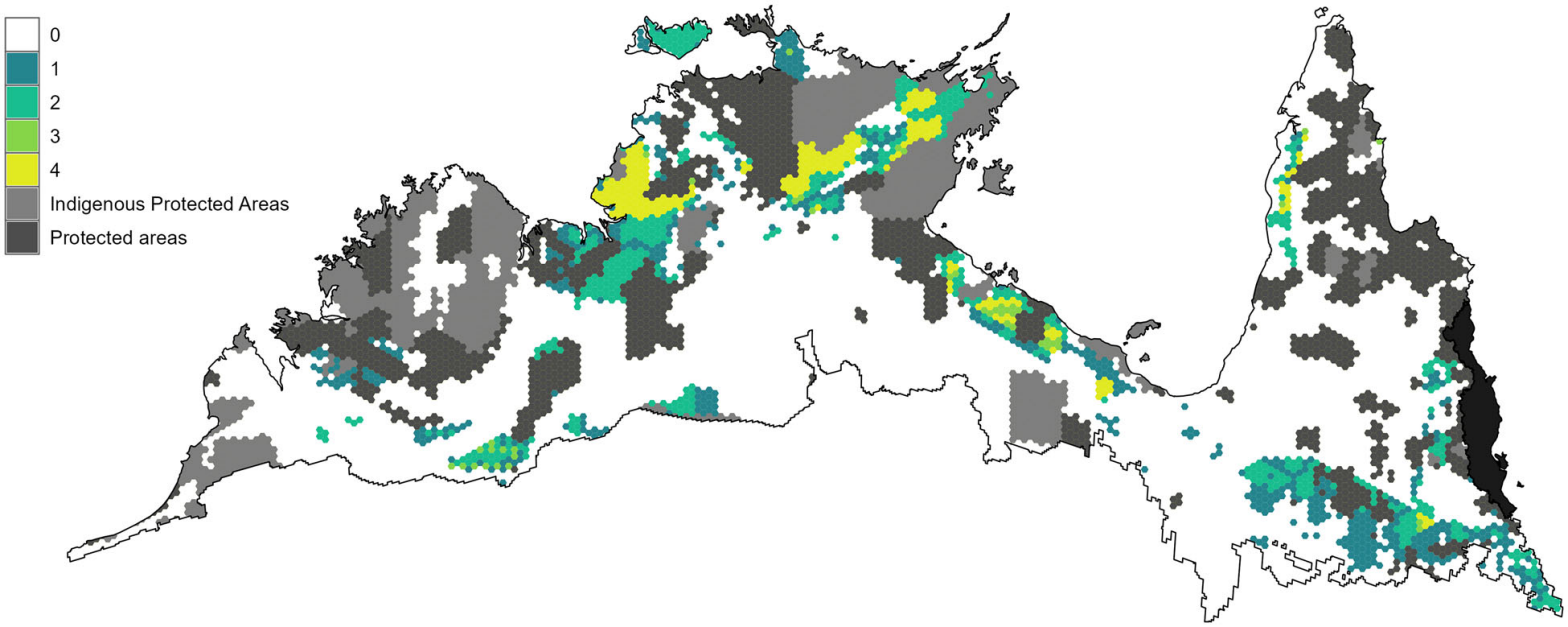
Selection frequency of planning units (PUs) summed across the 2 conservation objectives (expanding protected areas by adding to the existing network from any tenure and increasing the Indigenous protected area network through voluntary land declarations by traditional owners) and 2 protection criteria (mammal species currently considered threatened and mammals at high risk of extinction but not considered threatened) (numbers in the key, number of times a PU was selected across the 4 scenarios; black shading, Wet Tropics bioregion excluded from analyses; white, areas available for selection but not selected in any optimal solutions).

**FIGURE 4:**
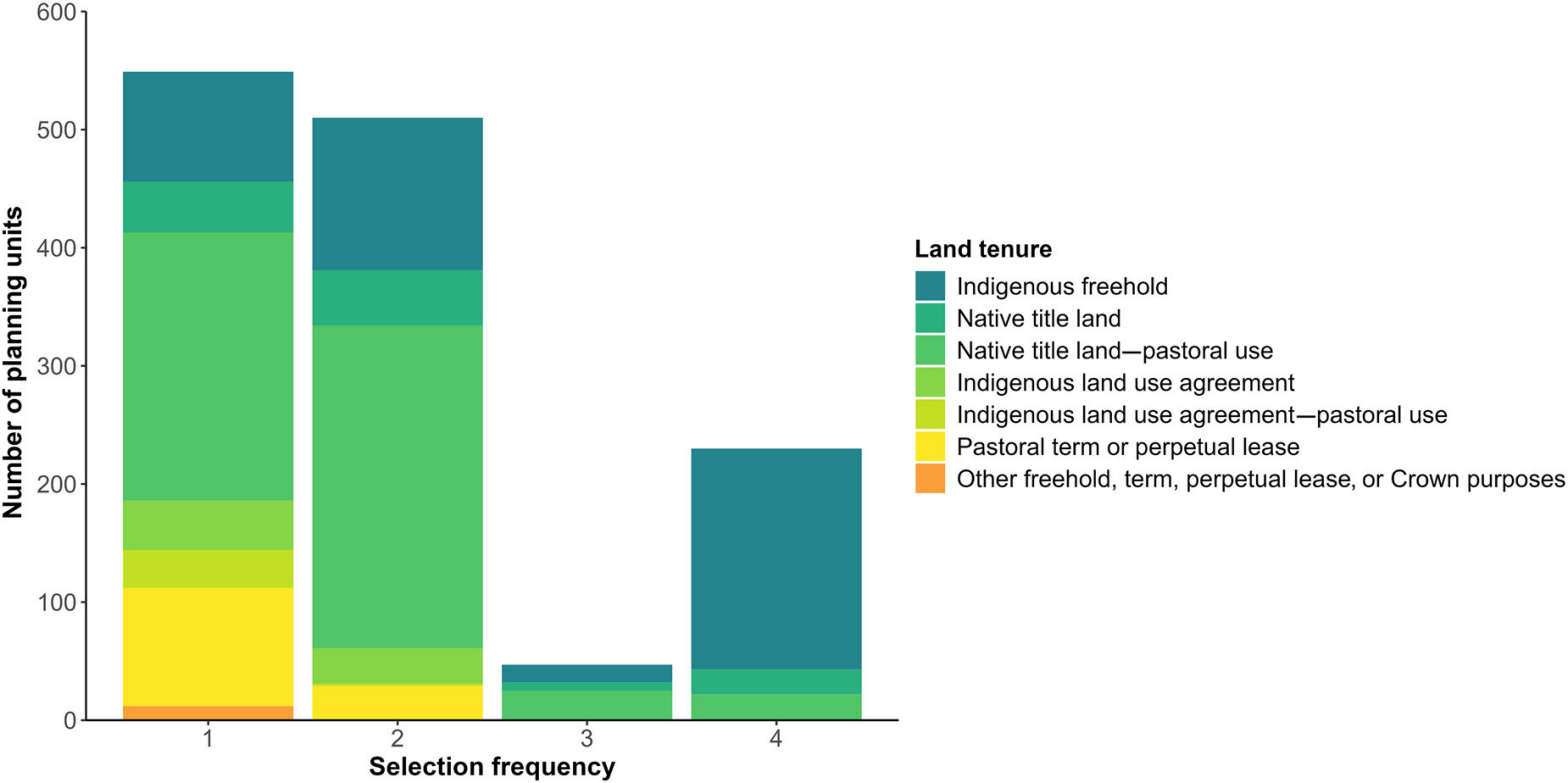
The number of planning units in each land tenure category selected in one, 2, 3, or all 4 of the conservation scenarios (i.e., combinations of objective and criteria). Objectives defined in Table 2 footnote, and criteria defined in “Systematic conservation planning.”

The spatial configuration of priority conservation areas optimized for different representation criteria (current and positive latent extinction risk) showed substantial variation in areas selected depending on the criterion (Figure 2) and influenced by constraints on land available for selection and proxy costs assigned to PUs. The PU selection frequency varied strongly by land tenure across all criteria: 81.3% of high‐priority PUs (i.e., PUs selected in all 4 scenarios) were located on Indigenous freehold land, mostly adjacent to existing PAs, highlighting the tendency for priority areas to enhance connectivity within the existing PA network (Figures 3 & 4).

When comparing the different biodiversity protection criteria and their associated species representation targets, we found that current extinction risk targets were achieved for the lowest total cost, smallest land area and boundary lengths, and lowest per‐unit cost (Table 2). These results reflected the strong spatial aggregation of threatened species across the AMT and the substantial existing coverage afforded to them by Australia's NRS. Thus, priority areas under the current risk criteria almost exclusively adjoined existing PAs (Figure 2a; Appendix S1). Probably for the same reason, the current extinction risk target exhibited slightly lower species coverage (i.e., the average proportion of the 128 AMT mammal species’ ranges secured in the optimal solution) (mean = 54.20%) than positive latent risk (mean = 55.15%) (Table 2).

**TABLE 2:** Results of optimal protected area configurations produced using the R package prioritizr for 4 conservation scenarios representing different combinations of objectives and protection criteria (current and positive latent risk).

Criterion	Objective^a^	Number of planning units	Proxy cost	Area (km^2^)	Boundary length (km)	Mean species coverage (%)
Current risk	1	608	70.08	89,001	30,913	54.23
	2	607	73.68	89,151	31,346	54.16
Positive latent risk	1	691	96.74	101,369	33,159	54.94
	2	684	117.45	100,219	31,899	55.35

^a^ Definitions: Objective 1, expansion of existing protected area network by adding any land tenure (e.g., pastoral leases, Indigenous land‐use agreements, Native Title land, Indigenous freehold, or other freehold, term, perpetual lease, or Crown purpose land); Objective 2, increase the size of existing protected area network through the voluntary declaration of Native Title and Indigenous freehold land as Indigenous protected areas by the traditional owners.

**FIGURE 5:**
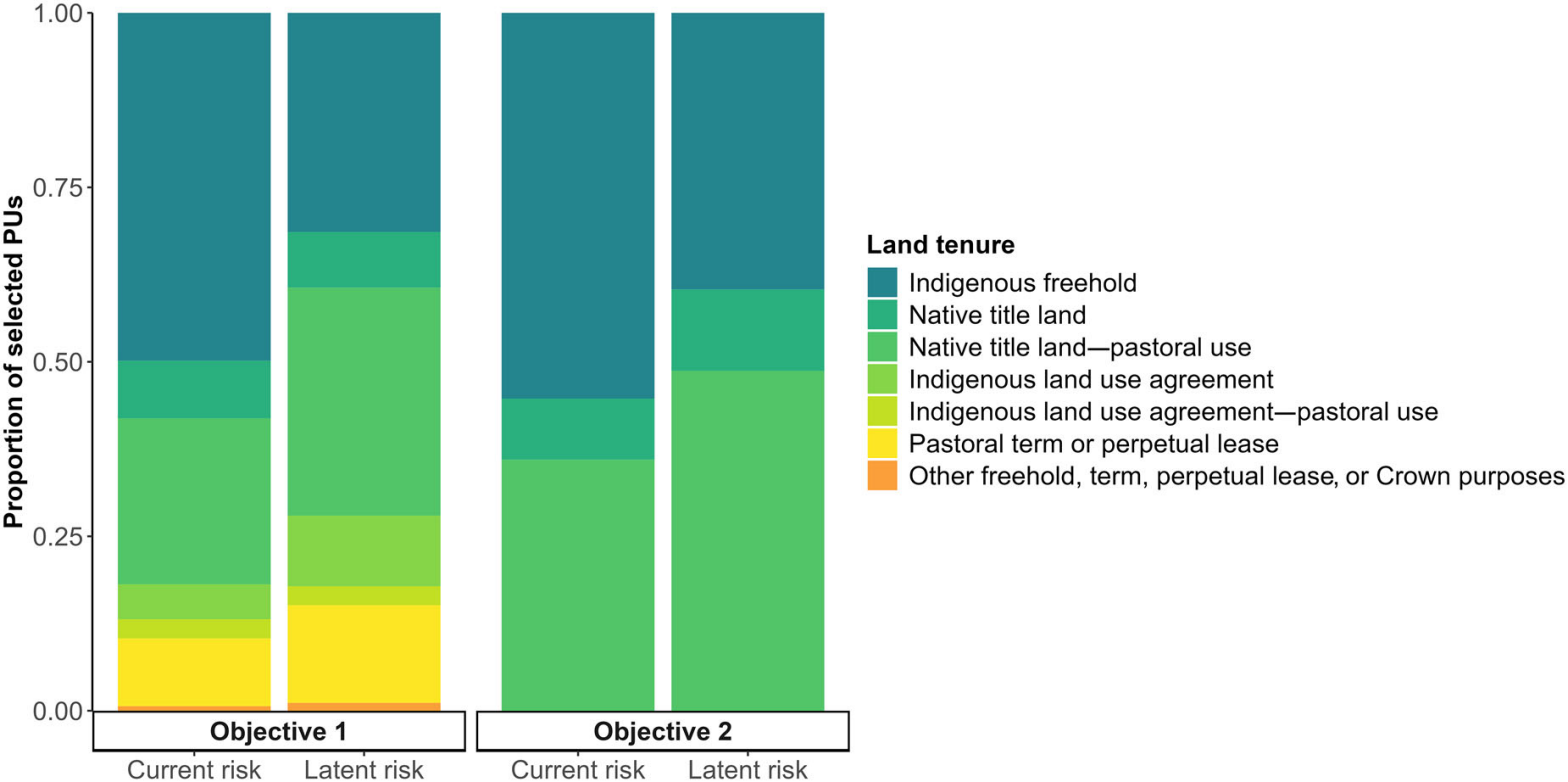
Proportion of selected planning units (PUs) in each land tenure category selected in the optimal protected area (PA) configurations for 4 conservation scenarios representing the prioritized protection of either currently threatened species (current risk) or mammal species with a positive latent extinction risk (Objective 1, PAs expanded by adding to the existing network from any tenure; Objective 2, Indigenous protected area network expanded through voluntary declaration of Native Title and Indigenous freehold land by traditional owners with other tenure excluded from selection).

Achieving the positive latent risk targets required, on average, approximately 13% more PUs compared with the current risk targets (Table 2). The average per‐unit cost under the positive latent risk criteria (mean = 0.16 cost units/PU) was higher than for current risk (mean = 0.12 cost units/PU) (Table 2). This cost difference likely reflected the increased dependence on pastoral land when prioritizing species with a positive latent risk (Figure 5). For Objective 1, 98 PUs were selected on pastoral term or perpetual leases under the positive latent risk criteria compared with 60 for the current risk criteria. Boundary length‐to‐area ratios, reflecting the compactness and connectivity of PAs, were lower under the positive latent risk criteria (mean = 0.32) than for current risk (mean = 0.36), indicating a marginally more cohesive optimal PA network when prioritizing positive latent extinction risk (Table 2).

There were substantial differences in target efficiency between the 2 PA design objectives. Expanding the IPA network through voluntary declaration of Native Title and Indigenous freehold land by traditional owners (Objective 2) was the costliest in terms of proxy cost, total land area, and per‐unit cost required to achieve targets (Table 2). For the current risk criteria, the difference was negligible, with approximately equal costs, area, boundary lengths, and numbers of PUs selected under both objectives (Table 2).

### Effect of design objectives and protection criteria on land tenure contributions

Expanding the NRS by adding to the existing PA network (Objective 1) required a combination of Indigenous freehold, Native Title, pastoral, and jointly managed tenure to achieve species representation targets under both criteria (Figure 5). For currently threatened species, 50.66% of PUs were on Indigenous freehold land, 24.18% on Native Title land coexisting with pastoral leases, and only 9.87% on pastoral leases alone (Figure 5). Under the positive latent risk criteria, approximately equal proportions of PUs were selected on Native Title land managed for grazing (33.14%) and Indigenous freehold land (31.84%), whereas 14.18% of PUs were located entirely on pastoral leases (Figure 5).

Under the second objective, which considered the scenario whereby traditional owners voluntarily declare Native Title and Indigenous freehold land to expand the IPA network, species’ representation targets for both criteria were met without relying on private, government, or Crown land (Figure 5). For the current risk criteria, 56.18% of PUs were on Indigenous freehold land and 36.57% were on Native Title land managed for grazing. For positive latent risk, 49.42% of PUs were on Native Title land under pastoral lease and 40.20% on Indigenous freehold land. Native Title land dependence was consistently low across scenarios (mean = 9.31 ± 1.72%), reflecting the low occurrence of nonpastoral land in the AMT.

## DISCUSSION

We addressed the interdependence of 2 emerging issues in conservation planning: the contribution of Indigenous lands to broader conservation systems (Morgan, 2021) and the need for a more proactive outlook that considers both current and potential future threats to biodiversity (Cardillo et al., 2023). We examined these issues in the context of expanding the PA network in the AMT with an SCP approach. Our prioritizations were not intended to prescribe formal additions to the NRS; rather, we sought to highlight areas where conservation actions may make the greatest contributions to protecting biodiversity, irrespective of tenure. Although our results support the potential contribution of Indigenous lands to optimal solutions within this framework, we make no claims or recommendations about methods of land management. Rather, by incorporating land tenure into the SCP process, our approach may help identify the appropriate communities or individuals for consultation and, where traditional owners wish to engage, support their early and informed participation in conservation planning. We recognize that the goals of traditional owners may differ from national‐ or global‐scale biodiversity conservation goals. Embedding Indigenous perspectives and self‐determination into conservation systems remains an urgent priority in many countries.

Indigenous approaches to land management and governance are associated with positive conservation outcomes. Recent work shows that tropical forests in protected Indigenous areas have higher ecological integrity and lower land‐use intensity than other areas (Sze et al., 2022). In many countries, Indigenous lands support vertebrate biodiversity that is comparable to, or greater than, that found in non‐Indigenous PAs (Schuster et al., 2019; Sze et al., 2024). Moreover, Australia's unfortunate record of mammal extinctions is suspected to be partly linked to the loss of traditional, Indigenous fire‐based land management in many regions (Johnson, 2006; Woinarski et al., 2015). Australia's guiding threatened species conservation framework explicitly plans for an increasingly important role for Indigenous communities and lands in conservation efforts (DCCEEW, 2022b). IPAs are being declared more rapidly than non‐Indigenous additions to the NRS, which is consistent with a recent government commitment of AU$231.5 million to the IPA program (Farr et al., 2016; Little et al., 2023). It is therefore timely to systematically examine the potential contributions of Indigenous lands compared with other tenures to ongoing biodiversity protection. Our results showed that Indigenous lands are central to achieving mammal protection targets in the AMT. However, the extent of their contribution varied depending on whether conservation priorities focused on current threats or anticipated future risks. Shifting the emphasis to species predicted to face high future extinction risks may offer a more effective long‐term strategy for safeguarding biodiversity (Cardillo et al., 2006), but it could also increase implementation costs due to greater reliance on pastoral and non‐freehold Indigenous lands relative to current risk targets (Figure 5).

### Comparative models and spatial patterns of extinction risk

Consistent with previous studies (Cardillo et al., 2008, 2023), our comparative models identified small geographic range size and large body mass as strong predictors of high extinction risk among AMT mammals. Hence, unthreatened species with small ranges, large body mass, or both tended to have higher latent risk, suggesting greater potential for future increases in threat status. Age of first reproduction also emerged as a significant predictor of extinction risk, independent of body mass, reflecting a slow life history and low population growth rates (Cardillo et al., 2006; van de Kerk et al., 2013). Among environmental variables, total and late dry season fire frequency were the strongest environmental predictors of threat status. Threat levels increased as the total number of years fire occurred in a species’ range increased, but threat levels decreased as late dry season fire frequency increased. Although this result seems contradictory, areas with higher late dry season fire frequency may experience lower overall fire frequency and contain more long‐unburned habitat because late dry season fires often follow extended periods of fire suppression. This interpretation is supported by studies indicating that overall fire frequency and extent are stronger predictors of faunal abundance in AMT savannas than fire seasonality and that long‐unburned areas enhance habitat complexity and biodiversity (Andersen, 2021; Andersen et al., 2005; Einoder et al., 2023; Evans & Russell‐Smith, 2020; Griffiths et al., 2015). Nonetheless, further investigation is needed to clarify the relationship between total and late dry season fire frequency.

Current extinction risk hotspots generally coincided with areas of high mammal species richness (Figure 1c; Appendix S7) and exhibited high coverage by the existing PA network (Figure 2a), highlighting a correlation between species richness, the number of threatened species, and the level of protection. In contrast, hotspots of latent risk were largely congruent with areas lacking protection (Figure 1a; Appendix S1). These patterns likely reflected the preferential establishment of PAs in regions of known biodiversity value, such as Kakadu National Park in the ecologically diverse Arnhem Plateau and Alligator River regions (DCCEEW, 2022a). However, part of this pattern may be artifactual because long‐term monitoring efforts are typically concentrated in PAs, increasing awareness of biodiversity and detection of population declines. Indeed, monitoring in the AMT has been largely confined to biodiversity hotspots in the Kimberley, Arnhem Plateau, and Cape York Peninsula (Preece & Fitzsimons, 2022), which supported higher concentrations of species currently listed as threatened (Figure 1c). In contrast, species with distributions outside PAs may be underrepresented on threatened species lists due to limited ecological and population data, resulting in elevated latent risk values.

### Systematic conservation planning

We found that the complementarity, representativeness, cost‐efficiency, and spatial distribution of priority conservation areas in the AMT varied depending on the protection criteria emphasized (current or latent extinction risk). Prioritizing currently threatened species was more cost‐effective, requiring less additional land to meet representation targets (Table 2). This result likely reflects the smaller geographic range sizes of threatened species and the strong spatial aggregation of species richness into relatively small hotspots, such as the eastern forests of Cape York Peninsula (Appendix S7). Consistent with previous studies (Astudillo‐Scalia & Albuquerque, 2020; Veach et al., 2017), protecting threatened mammal species, often concentrated in hotspots of species richness (Figure 1c; Appendix S7), was less representative of the broader AMT mammal assemblage (Table 2).

Protecting species with high latent risk required greater PA expansion than for currently threatened species, making this choice more costly. However, prioritizing positive latent risk species improved overall species representation and enhanced complementarity with Australia's NRS (Table 2). Although the AMT reserve network's high coverage of biodiversity hotspots may have been most cost‐effective initially, expanding protection to species likely to become threatened in the future could mitigate threats, reduce long‐term recovery costs, and improve NRS complementarity and representativeness (Cardillo et al., 2006). Securing land for high latent risk species may additionally slow environmental degradation and increase species persistence (Lindenmayer et al., 2007). These findings highlight the need for a more proactive, inclusive approach to conservation planning in Australia to minimize future costs and slow population declines (Walsh et al., 2012). Using SCP to evaluate prioritization scenarios may help decision makers move from ad hoc additions to the PA network to solutions that maximize biodiversity outcomes and minimize costs (Hanson et al., 2024; Sarkar et al., 2008).

Our method represents one of many approaches used in SCP, each suited to different objectives and contexts. For example, species distribution models are well‐suited to identifying fine‐scale spatial priorities for urgent interventions targeting a small number of threatened species (Morán‐Ordóñez et al., 2025). In contrast, global criteria‐based frameworks used for conservation planning, such as key biodiversity areas, prioritize areas based on irreplaceability and vulnerability criteria, independent of existing PA coverage (Langhammer et al., 2018; Plumptre et al., 2025). Although our approach may not suit all contexts, it is particularly valuable for planning across large, multitenure landscapes where efficient and representative PA networks are needed to address both current and emerging conservation priorities. Ultimately, the choice of method should reflect the specific goals, spatial scale, and data availability of the planning context.

Prioritizations are subject to several limitations. Prioritization tools, such as prioritizr (Hanson et al., 2022) and Marxan (Ball et al., 2011), depend on user‐defined inputs, such as representation targets, PU costs, and area constraints (Schuster et al., 2020). Rather than simply selecting hotspots of the emphasized protection criterion, these algorithms identify priority areas in a systematic manner sensitive to input variations. In our study, the lack of accessible data reflecting land valuations and legal costs for IPA declarations constrained accurate cost representation in prioritization scenarios, and more reliable data may yield different results. Integrating threat abatement costs in prioritization assessments is also challenging, potentially constraining the selection of cost‐efficient solutions. Nonetheless, prioritization remains an important conservation tool that can be updated as priorities shift and new information becomes available (Wilson, 2023).

### Contribution of land tenures to AMT mammal conservation

The inclusion of Indigenous lands was crucial for achieving conservation goals in the AMT, whether emphasizing the protection of both currently threatened species or species predicted to become threatened (Figure 5). Notably, the highest priority areas for all scenarios required minimal contribution from pastoral land, including Native Title land jointly managed for grazing (Figure 4). This finding has significant implications for conservation management in the AMT, illustrating that opportunity costs associated with the beef cattle grazing industry can be minimized when allocating PAs. Omitting pastoral land from the PA network additionally reduces the costs of restoring areas degraded by overgrazing, invasive grasses, and frequent fires (Woinarski et al., 2013). Regardless of land tenure type, improving ecological condition by reducing livestock densities and reinstating appropriate fire regimes should be considered essential for the recovery of native mammal species outside PAs (Mappin et al., 2022; Woinarski et al., 2013). It is possible for pastoral land to simultaneously support livelihoods and convey benefits to biodiversity, as demonstrated by successful collaborative conservation projects on pastoral land (Legge et al., 2011; Yibarbuk et al., 2001).

The total proxy costs to achieve species representation targets were higher under the second objective of expanding the IPA network through the voluntary declaration of Indigenous lands by traditional owners, though the cost difference was negligible under the current risk criterion (Table 2). Increased costs under the second objective were likely due to more PUs selected in the Einasleigh Uplands, Victoria Bonaparte, and Central Kimberley bioregions (Figure 2; Appendix S1), where higher median income and anthropogenic development associated with the profitable beef industry contribute to elevated proxy costs (Chudleigh et al., 2019; Appendix S2). Although these costs were significant, the voluntary addition of Native Title land would be expected to offset land acquisition expenses, making the second objective potentially more cost‐effective if the relevant traditional owners desire to contribute their land to the IPA network. This potential reduction in PU costs was not factored into the prioritization scenarios to avoid introducing bias in selecting Indigenous‐managed lands. There are also considerable consultation and legal expenses in IPA establishment to certify that traditional owners approve all aspects of the IPA agreement and consent to committing their land to the NRS in perpetuity (Farr et al., 2016; Hill et al., 2011), further complicating cost analyses. Nonetheless, the socioeconomic, biocultural, and environmental benefits conferred by expanding the IPA network, including through increased Indigenous employment, connection to country, and reinstatement of traditional land management practices, are substantial and worth investing in for the future (Farr et al., 2016).

Although we did not incorporate culturally significant species, places, or ecological communities into prioritizations, we recognize their importance in conservation planning on Indigenous lands (Goolmeer et al., 2022). Integrating these features in a meaningful and culturally appropriate manner requires detailed consultation with traditional owners. Our approach is intended as an initial step toward identifying priority areas for PA expansion by accounting for the need to anticipate future threats to biodiversity and the costs and benefits of allocating different land tenures for conservation. Where broadscale analyses identify Indigenous lands as important for achieving biodiversity targets, more detailed, fine‐scale planning should be led by Indigenous communities with their free, prior, and informed consent. Indigenous‐led planning enables Indigenous communities to define aspirations for their lands, regardless of formal tenure status, and facilitates the application of Indigenous land management practices, such as cultural burning, as part of broader conservation management strategies (Hill et al., 2013). Ultimately, SCP on Indigenous lands must be embedded in governance structures that respect Indigenous knowledge systems, customary law, and decision‐making authority.

Achieving global biodiversity goals increasingly requires conservation strategies that recognize the importance of incorporating diverse land tenures and proactively addressing emerging threats (CBD, 2018; Fitzsimons et al., 2024). Our findings demonstrate how land tenure dependence can shift when prioritizing currently threatened species versus species likely to become threatened in the future. Indigenous and pastoral lands are likely to play an increasingly important role in achieving conservation outcomes, though their relative contributions will depend on how future conservation priorities are framed. IPAs offer a pathway to enhance biodiversity outcomes through traditional land management (Legge et al., 2011) and provide cobenefits for Indigenous communities through increased employment, intergenerational knowledge transfer, and cultural connection to traditional lands (Little et al., 2023). Building protected area networks that reflect both conservation priorities and Indigenous self‐determination will be essential to creating equitable, effective conservation systems capable of mitigating biodiversity declines. Importantly, the addition of Indigenous lands to conservation areas must only occur at the initiation of the Indigenous people who are the traditional custodians of the land.

## Supporting information



Supplementary Information
