# Supplementary material for: Land tenure contributions to protected area growth under alternative conservation targets in the Australian monsoon tropics
Source: Conserv Biol. 2025 Sep 9;40(1):e70143. doi: 10.1111/cobi.70143 (PMC12856816; doi:10.1111/cobi.70143)
Supplement: Supplementary file 1 — Supplementary Information [file COBI-40-e70143-s001.docx]

**APPENDIX S1**

Map of northern Australia depicting the Interim Biogeographical Regionalization for Australia version 7.0 (IBRA) bioregions (DCCEEW, 2021). The green area represents the Australian Monsoonal Tropics phytogeographic sub-region (Brundrett, 2017) used as the focal study area. The Wet Tropics bioregion, shaded dark green, was excluded from analysis due to comprising a highly unique and endemic flora and fauna.


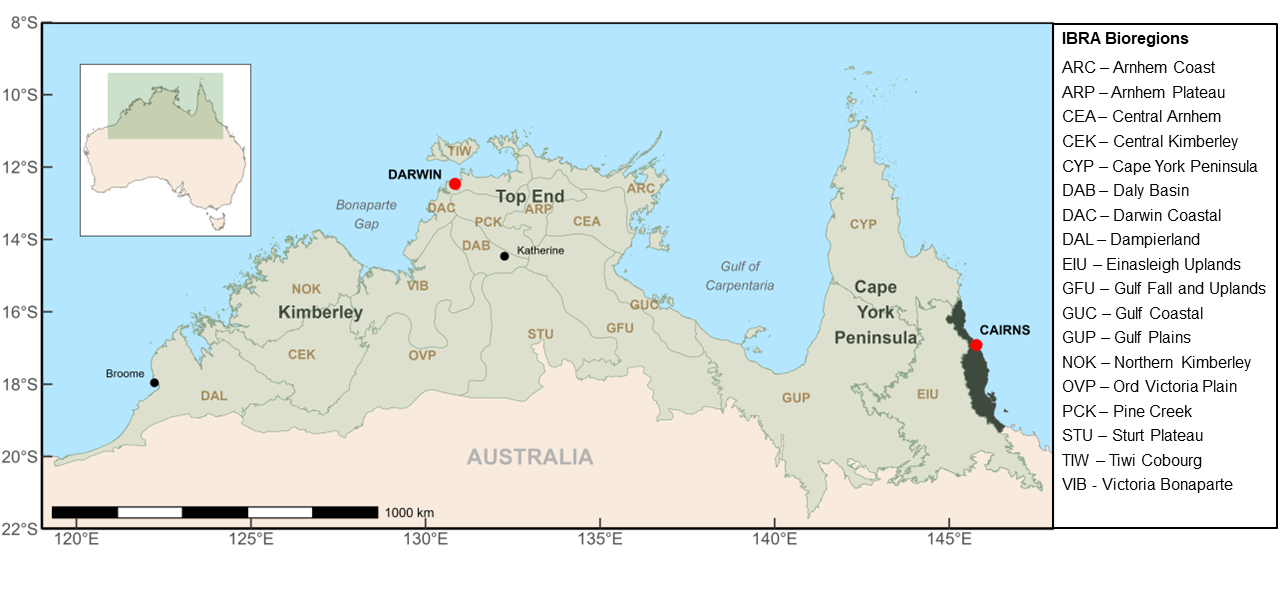


**APPENDIX S2**

Maps of (a) median employee income for local government areas (ABS, 2018) and (b) human influence index, which classifies the degree of human impact on a 0-61 point scale (Sanderson et al., 2002), which were combined to create (c) the final proxy cost layer used to identify optimal cost-efficient protected area configurations for mammal species in the Australian Monsoonal Tropics.

**(a)**

**
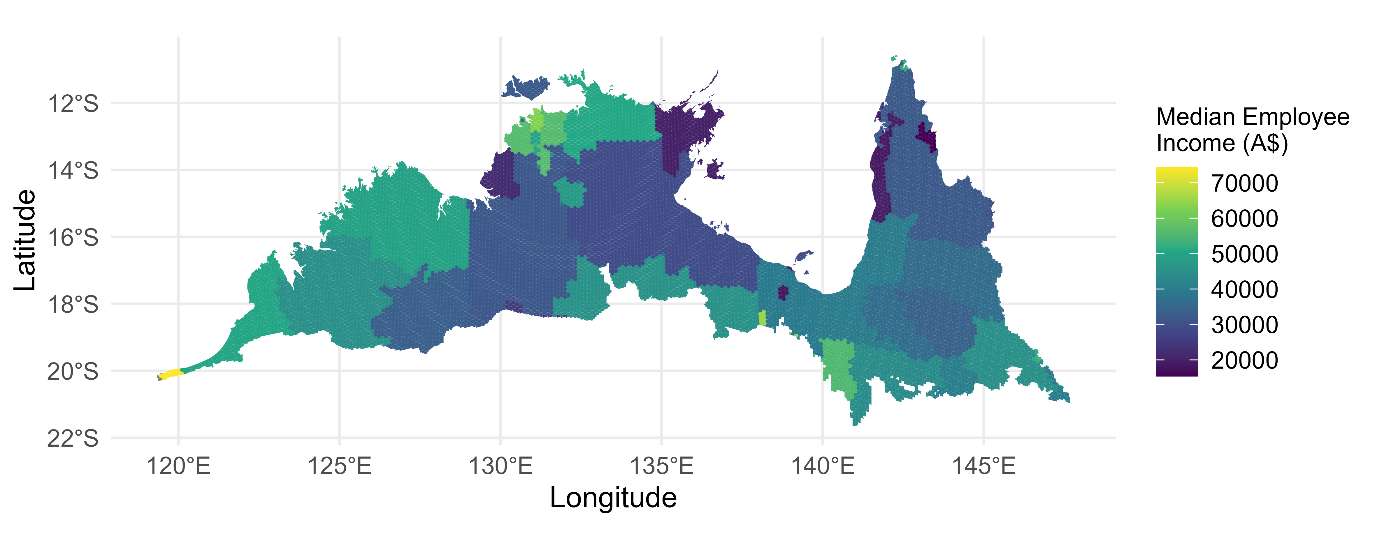
**

**(b)**

**
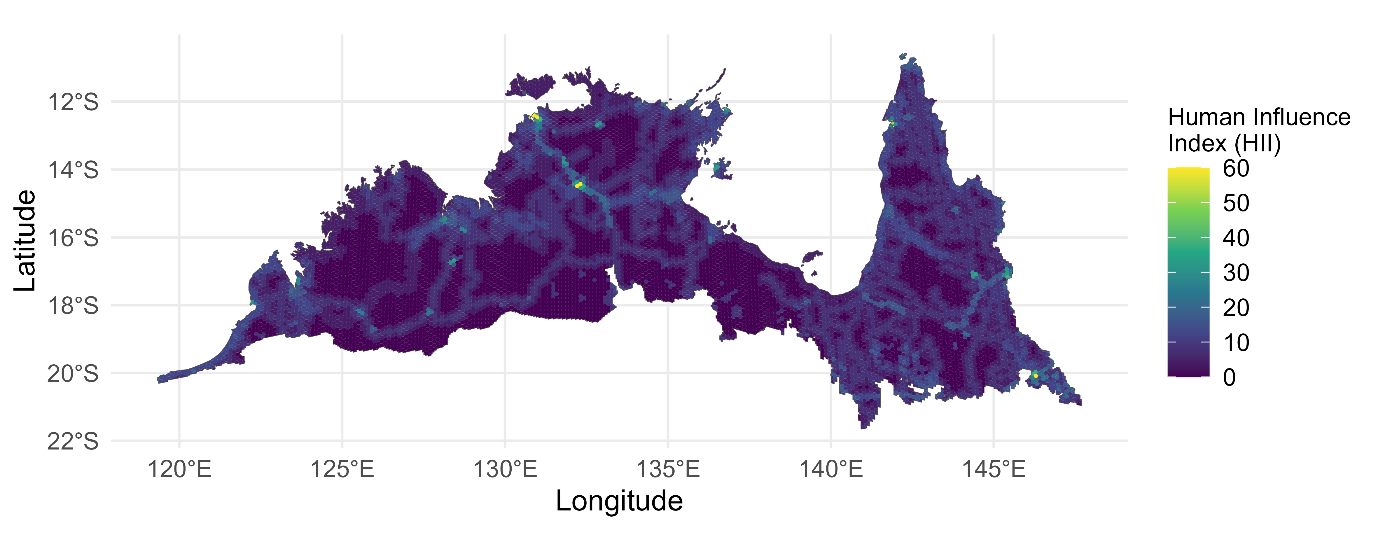
**

**(c)**

**
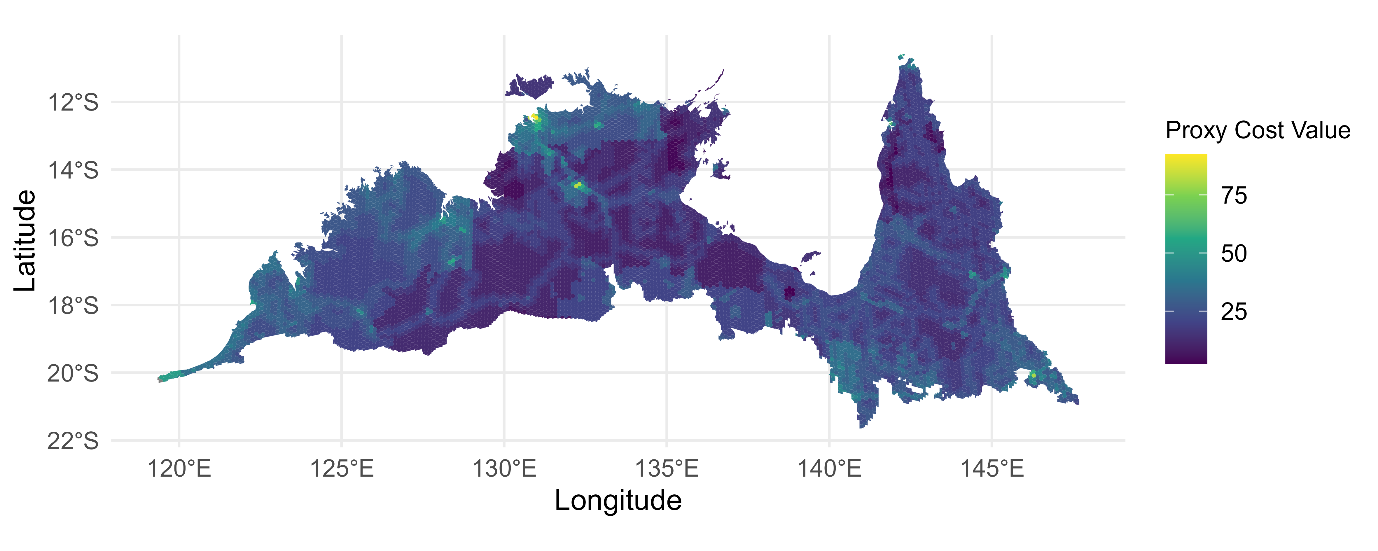
**

**APPENDIX S3**

Map of land tenure categories assigned to planning units in the Australian Monsoonal Tropics (AMT).


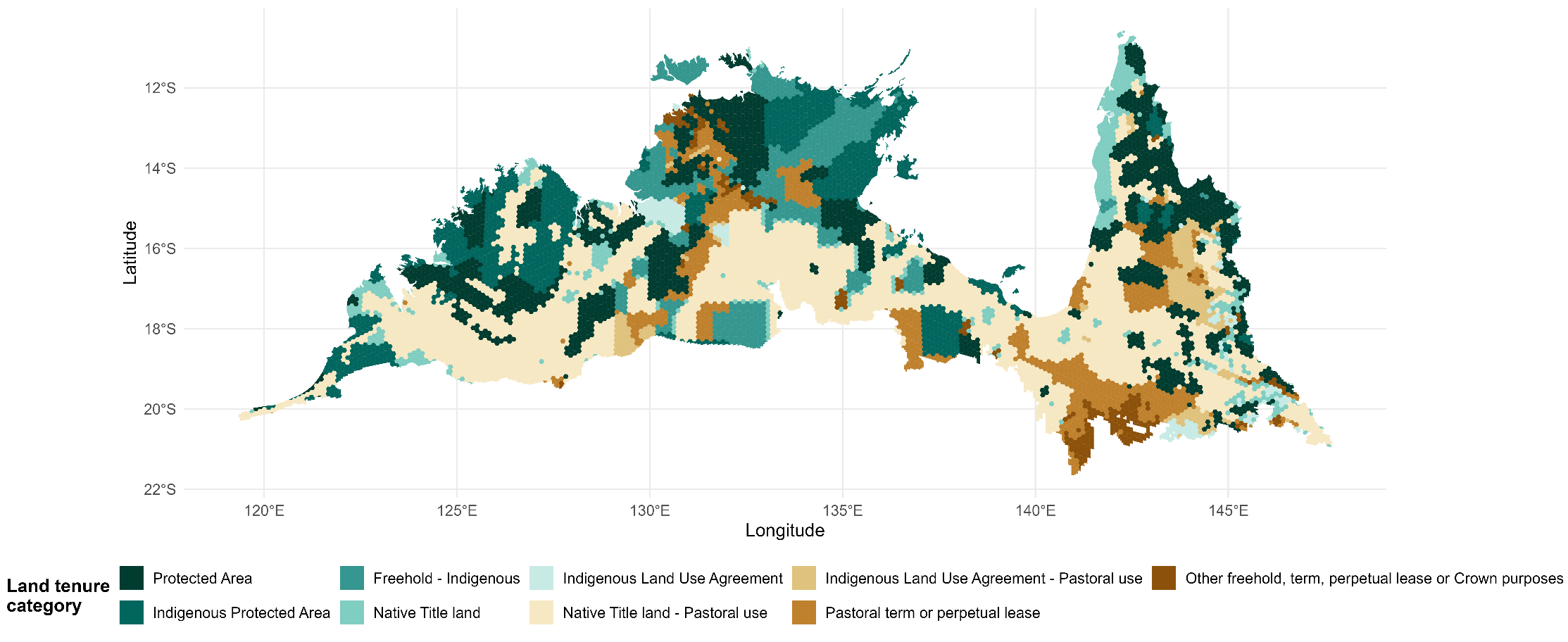


**APPENDIX S4**

Covariate values for 128 mammal species native to the Australian Monsoonal Tropics (AMT) included in phylogenetic generalized least squares (GLS) models to predict species’ latent extinction risk. Values are presented for each covariate prepared for use in the models. Generation length and precipitation of the driest quarter were excluded from the full and minimum adequate models due to collinearity issues.

| Scientific Name | Geographic range size (km^2^) ^*^ | % range overlap with AMT | Mean adult body mass (g) ^*^ | Age of first reproduction (days) ^*^ | Mean litter size | Mean no. litters per year^†^ | Generation length (days) | % range overlap with red fox distribution^†^ | % range overlap with cane toad distribution^†^ | Mean fire frequency^*^ | Mean late dry season fire frequency^*^ | Mean BIO8^a^ | Mean BIO17^b^ | Mean HII^c^ |
| --- | --- | --- | --- | --- | --- | --- | --- | --- | --- | --- | --- | --- | --- | --- |
| *Acrobates pygmaeus* | 1199224.83 | 10.86 | 13.00 | 212.91 | 3.00 | 2.00 | 997.97 | 88.74 | 62.03 | 6.50 | 4.77 | 26.78 | 25.12 | 5.42 |
| *Aepyprymnus rufescens* | 566140.53 | 15.70 | 2820.00 | 319.37 | 1.02 | 3.00 | 1327.59 | 89.97 | 99.80 | 2.38 | 1.78 | 26.33 | 27.64 | 4.54 |
| *Antechinus adustus* | 4920.50 | 15.54 | 30.50 | 317.18 | 6.00 | 1.00 | 615.65 | 0.00 | 100.00 | 3.87 | 3.19 | 24.18 | 52.70 | 5.20 |
| *Antechinus bellus* | 20506.45 | 99.99 | 36.37 | 426.83 | 10.08 | 1.00 | 622.12 | 0.00 | 0.00 | 13.31 | 3.51 | 28.21 | 7.37 | 2.92 |
| *Antechinus leo* | 4344.47 | 99.99 | 70.09 | 427.83 | 9.10 | 1.00 | 533.43 | 0.00 | 0.00 | 1.09 | 1.06 | 26.82 | 53.00 | 6.00 |
| *Cercartetus caudatus* | 24912.86 | 30.60 | 23.05 | 465.00 | 2.37 | 2.00 | 668.87 | 0.00 | 100.00 | 3.42 | 2.78 | 24.65 | 48.15 | 5.75 |
| *Chaerephon jobensis* | 2266001.00 | 58.76 | 20.71 | 379.30 | 1.01 | 1.66 | 1498.17 | 30.48 | 58.31 | 6.75 | 4.21 | 28.93 | 10.53 | 3.04 |
| *Chalinolobus gouldii* | 7361508.46 | 14.61 | 15.87 | 457.18 | 1.71 | 0.75 | 2264.72 | 81.59 | 40.52 | 6.60 | 3.82 | 29.25 | 9.55 | 2.76 |
| *Chalinolobus nigrogriseus* | 1396350.66 | 80.56 | 8.79 | 419.55 | 1.50 | 1.03 | 1718.96 | 22.29 | 65.32 | 7.23 | 4.38 | 28.69 | 10.58 | 3.25 |
| *Conilurus penicillatus* | 343479.24 | 99.98 | 159.51 | 156.03 | 2.00 | 3.32 | 547.50 | 3.64 | 28.41 | 8.53 | 3.95 | 28.84 | 7.13 | 3.42 |
| *Dactylopsila trivirgata* | 36727.38 | 53.02 | 406.71 | 533.66 | 1.52 | 1.10 | 1658.86 | 0.00 | 68.54 | 3.71 | 3.23 | 26.27 | 48.97 | 5.71 |
| *Dasyurus hallucatus* | 329576.83 | 40.44 | 564.50 | 374.16 | 6.60 | 1.00 | 731.39 | 17.46 | 26.85 | 9.39 | 3.73 | 27.74 | 14.16 | 3.99 |
| *Dendrolagus bennettianus* | 3511.65 | 35.06 | 9581.00 | 841.68 | 1.00 | 1.10 | 3252.33 | 0.00 | 100.00 | 3.82 | 3.22 | 25.79 | 39.96 | 6.48 |
| *Dendrolagus lumholtzi* | 7377.80 | 11.42 | 6649.97 | 911.46 | 1.00 | 1.00 | 3527.49 | 0.00 | 100.00 | 3.55 | 3.03 | 24.30 | 54.40 | 7.00 |
| *Echymipera rufescens* | 54156.82 | 100.00 | 1150.74 | 367.51 | 2.20 | 1.50 | 866.58 | 0.00 | 0.00 | 8.14 | 5.73 | 27.07 | 19.72 | 5.48 |
| *Hipposideros ater* | 991321.42 | 94.31 | 7.13 | 380.20 | 0.99 | 1.64 | 1469.85 | 4.53 | 57.28 | 7.56 | 4.46 | 28.62 | 9.84 | 3.28 |
| *Hipposideros cervinus* | 34156.33 | 99.59 | 8.51 | 707.94 | 1.00 | 1.00 | 2197.64 | 0.00 | 0.00 | 6.71 | 5.12 | 27.03 | 26.83 | 5.88 |
| *Hipposideros diadema* | 112995.28 | 85.58 | 46.90 | 442.89 | 1.00 | 1.70 | 1749.53 | 0.00 | 63.32 | 5.56 | 4.11 | 26.74 | 23.60 | 4.84 |
| *Hipposideros semoni* | 72516.41 | 93.87 | 14.00 | 761.30 | 1.03 | 1.00 | 2372.50 | 6.02 | 23.91 | 6.99 | 5.05 | 27.29 | 23.32 | 5.22 |
| *Hipposideros stenotis* | 487483.91 | 97.32 | 12.00 | 753.48 | 1.02 | 1.00 | 2313.76 | 2.29 | 40.38 | 8.59 | 4.28 | 28.93 | 6.99 | 3.16 |
| *Hydromys chrysogaster* | 3032691.50 | 25.50 | 738.09 | 158.00 | 3.80 | 2.12 | 824.90 | 75.03 | 67.72 | 6.92 | 4.37 | 28.50 | 12.25 | 3.90 |
| *Hypsiprymnodon moschatus* | 10445.73 | 10.44 | 534.25 | 365.00 | 2.04 | 1.10 | 894.25 | 0.00 | 100.00 | 3.51 | 2.74 | 24.19 | 53.84 | 4.68 |
| *Isoodon macrourus* | 1167357.17 | 51.08 | 1551.00 | 148.00 | 3.57 | 2.66 | 623.75 | 51.03 | 66.43 | 8.62 | 4.72 | 27.99 | 12.53 | 3.77 |
| *Isoodon obesulus* | 200977.52 | 6.27 | 717.00 | 186.10 | 2.53 | 2.20 | 683.31 | 57.46 | 0.12 | 3.06 | 2.89 | 27.02 | 40.54 | 6.78 |
| *Lagorchestes conspicillatus* | 1880580.49 | 61.55 | 2740.51 | 421.43 | 1.00 | 1.71 | 1100.22 | 39.45 | 77.18 | 6.56 | 4.12 | 29.03 | 10.07 | 2.84 |
| *Leggadina forresti* | 1981304.49 | 5.01 | 21.94 | 122.80 | 3.50 | 3.29 | 544.88 | 98.55 | 65.63 | 1.65 | 1.57 | 30.02 | 13.02 | 3.18 |
| *Leggadina lakedownensis* | 1322193.11 | 66.54 | 17.50 | 123.89 | 3.00 | 3.31 | 537.79 | 17.32 | 52.03 | 6.96 | 4.08 | 28.83 | 10.55 | 2.76 |
| *Macroderma gigas* | 1153385.94 | 64.46 | 139.00 | 456.57 | 1.00 | 1.00 | 1460.00 | 22.32 | 46.25 | 7.31 | 3.89 | 28.60 | 11.03 | 3.34 |
| *Macroglossus minimus* | 467879.79 | 98.03 | 16.10 | 212.91 | 0.99 | 3.00 | 1139.14 | 0.00 | 27.21 | 8.95 | 5.11 | 28.16 | 10.05 | 4.09 |
| *Macropus giganteus* | 2229661.59 | 10.47 | 41454.95 | 812.60 | 1.00 | 1.00 | 3170.27 | 91.70 | 68.25 | 4.69 | 3.86 | 27.51 | 20.15 | 3.65 |
| *Macrotis lagotis* | 1079691.62 | 12.06 | 1269.75 | 193.48 | 1.75 | 4.00 | 1183.87 | 73.89 | 27.86 | 4.86 | 3.73 | 30.39 | 8.52 | 2.73 |
| *Melomys burtoni* | 452198.46 | 68.00 | 62.00 | 149.01 | 2.50 | 3.22 | 631.35 | 29.72 | 43.75 | 8.90 | 4.78 | 27.96 | 13.49 | 4.67 |
| *Melomys capensis* | 31043.14 | 100.00 | 70.60 | 210.01 | 2.20 | 1.30 | 764.16 | 0.00 | 0.00 | 6.50 | 5.55 | 27.03 | 27.25 | 6.61 |
| *Melomys cervinipes* | 216584.54 | 6.06 | 82.60 | 210.01 | 2.35 | 1.30 | 771.85 | 84.30 | 98.65 | 3.87 | 3.22 | 25.92 | 44.90 | 6.41 |
| *Mesembriomys gouldii* | 235643.32 | 89.62 | 900.00 | 95.04 | 1.96 | 4.00 | 712.23 | 11.71 | 40.73 | 7.56 | 4.82 | 27.20 | 18.90 | 5.03 |
| *Mesembriomys macrurus* | 34072.88 | 100.00 | 268.50 | 226.29 | 2.00 | 2.07 | 846.53 | 0.00 | 0.00 | 9.12 | 5.08 | 28.48 | 11.32 | 3.29 |
| *Miniopterus australis* | 463925.97 | 44.25 | 7.40 | 706.54 | 1.00 | 1.00 | 1845.23 | 59.43 | 87.75 | 4.82 | 3.69 | 26.95 | 23.29 | 4.51 |
| *Murina florium* | 54391.75 | 67.51 | 4.41 | 461.06 | 1.25 | 1.02 | 1945.38 | 0.73 | 89.79 | 3.11 | 2.50 | 25.69 | 35.19 | 5.07 |
| *Myotis macropus* | 1322380.29 | 53.67 | 10.41 | 303.71 | 1.00 | 2.50 | 1356.01 | 46.46 | 48.68 | 8.35 | 4.53 | 28.45 | 10.69 | 3.76 |
| *Notoryctes caurinus* | 451104.14 | 17.44 | 34.00 | 537.32 | 1.50 | 1.68 | 1868.76 | 62.22 | 9.30 | 4.24 | 3.07 | 30.69 | 8.24 | 2.65 |
| *Nyctimene robinsoni* | 349546.34 | 43.47 | 48.83 | 769.56 | 1.00 | 1.00 | 2224.74 | 54.22 | 78.90 | 6.48 | 4.69 | 26.82 | 22.99 | 5.17 |
| *Nyctophilus arnhemensis* | 700919.89 | 99.17 | 6.83 | 370.81 | 2.00 | 1.08 | 1548.15 | 0.00 | 46.47 | 7.99 | 4.23 | 29.03 | 7.71 | 3.05 |
| *Nyctophilus bifax* | 398874.63 | 52.31 | 10.61 | 349.75 | 2.00 | 1.66 | 1338.50 | 47.39 | 77.91 | 6.74 | 5.01 | 27.03 | 20.37 | 4.65 |
| *Nyctophilus geoffroyi* | 7264622.40 | 14.54 | 8.00 | 358.55 | 1.78 | 1.14 | 1456.59 | 81.00 | 39.45 | 6.62 | 3.86 | 29.33 | 8.70 | 2.69 |
| *Nyctophilus gouldi* | 1498214.13 | 7.06 | 11.32 | 349.75 | 1.77 | 1.69 | 1346.39 | 95.93 | 71.40 | 2.06 | 1.62 | 26.77 | 27.91 | 4.06 |
| *Nyctophilus walkeri* | 396100.71 | 98.76 | 4.44 | 362.34 | 1.80 | 1.27 | 1501.46 | 0.73 | 31.78 | 8.99 | 4.31 | 28.81 | 6.71 | 3.38 |
| *Onychogalea unguifera* | 950028.45 | 93.44 | 5800.00 | 420.58 | 1.00 | 1.47 | 1367.35 | 6.87 | 72.79 | 6.64 | 4.14 | 29.33 | 7.82 | 2.55 |
| *Ornithorhynchus anatinus* | 899738.21 | 2.40 | 1225.00 | 730.00 | 2.00 | 1.10 | 2878.76 | 89.85 | 59.98 | 3.48 | 2.51 | 26.25 | 42.19 | 6.65 |
| *Perameles nasuta* | 410003.33 | 3.60 | 720.26 | 121.66 | 2.57 | 2.92 | 679.14 | 92.18 | 63.74 | 3.41 | 2.53 | 25.51 | 47.63 | 5.21 |
| *Petauroides volans* | 632911.65 | 7.97 | 1141.00 | 730.00 | 1.00 | 1.00 | 2106.05 | 96.34 | 81.88 | 2.24 | 1.62 | 26.16 | 34.08 | 5.03 |
| *Petaurus breviceps* | 1893097.27 | 37.58 | 127.55 | 403.02 | 2.00 | 1.30 | 1969.16 | 60.13 | 54.77 | 8.04 | 4.44 | 28.27 | 11.49 | 3.71 |
| *Petaurus norfolcensis* | 787851.19 | 17.32 | 272.50 | 405.45 | 1.57 | 1.56 | 1677.08 | 84.87 | 83.38 | 6.40 | 4.54 | 26.98 | 18.65 | 4.25 |
| *Petrogale assimilis* | 208612.65 | 47.06 | 4618.88 | 626.25 | 1.00 | 1.49 | 1838.66 | 93.11 | 100.00 | 2.79 | 2.49 | 28.40 | 22.07 | 3.43 |
| *Petrogale brachyotis* | 220346.44 | 99.99 | 4000.00 | 570.00 | 1.00 | 1.50 | 1771.79 | 0.00 | 27.28 | 9.20 | 4.21 | 28.91 | 7.42 | 2.91 |
| *Petrogale burbidgei* | 17019.88 | 99.99 | 1258.00 | 573.46 | 1.00 | 1.40 | 1854.30 | 0.00 | 0.00 | 9.64 | 5.15 | 28.02 | 13.38 | 2.73 |
| *Petrogale concinna* | 81931.26 | 100.00 | 1324.10 | 508.75 | 1.00 | 1.00 | 1880.16 | 0.00 | 0.00 | 9.98 | 4.46 | 28.29 | 8.50 | 2.49 |
| *Petrogale godmani* | 36127.43 | 98.24 | 4750.00 | 571.15 | 1.01 | 1.54 | 1755.66 | 0.00 | 81.51 | 6.18 | 4.88 | 27.25 | 22.46 | 4.46 |
| *Petrogale mareeba* | 19941.20 | 96.21 | 4150.00 | 581.92 | 1.04 | 1.59 | 1782.75 | 0.00 | 100.00 | 3.15 | 2.36 | 25.81 | 20.70 | 5.12 |
| *Petrogale purpureicollis* | 80360.70 | 8.95 | 5283.94 | 570.00 | 1.00 | 1.48 | 1897.46 | 91.88 | 100.00 | 4.13 | 3.47 | 30.08 | 11.87 | 1.20 |
| *Petrogale sharmani* | 16921.69 | 78.31 | 4250.00 | 582.17 | 1.04 | 1.59 | 1789.85 | 1.44 | 100.00 | 2.10 | 1.65 | 25.58 | 40.03 | 4.59 |
| *Petropseudes dahli* | 198518.74 | 91.20 | 1877.03 | 72.00 | 1.00 | 2.00 | 1006.26 | 7.47 | 58.74 | 8.01 | 4.19 | 28.98 | 8.18 | 2.04 |
| *Phalanger mimicus* | 5789.56 | 99.99 | 2293.75 | 388.40 | 2.00 | 1.52 | 1254.34 | 0.00 | 0.00 | 1.69 | 1.60 | 26.70 | 46.64 | 5.34 |
| *Phascogale pirata* | 39951.40 | 99.99 | 181.50 | 401.27 | 6.60 | 1.00 | 878.35 | 0.00 | 0.27 | 12.45 | 3.80 | 28.18 | 7.49 | 2.87 |
| *Phascogale tapoatafa* | 270456.71 | 18.12 | 157.00 | 401.27 | 6.60 | 1.00 | 878.35 | 76.27 | 42.32 | 7.79 | 4.56 | 27.89 | 21.45 | 3.49 |
| *Phascolarctos cinereus* | 1100687.77 | 8.85 | 5646.87 | 730.00 | 1.01 | 1.00 | 2746.44 | 95.10 | 80.02 | 2.24 | 1.73 | 26.45 | 27.35 | 4.09 |
| *Phoniscus papuensis* | 398268.75 | 26.85 | 6.32 | 449.40 | 1.22 | 1.01 | 1911.45 | 70.85 | 81.90 | 6.01 | 4.52 | 26.73 | 27.36 | 5.54 |
| *Pipistrellus adamsi* | 276062.70 | 99.99 | 3.34 | 114.84 | 1.92 | 3.00 | 1248.15 | 0.00 | 3.67 | 9.97 | 4.76 | 28.21 | 8.59 | 4.13 |
| *Pipistrellus westralis* | 187156.10 | 99.99 | 3.34 | 114.84 | 1.92 | 3.00 | 1252.67 | 0.00 | 18.34 | 7.86 | 4.32 | 28.99 | 7.73 | 4.49 |
| *Planigale ingrami* | 616901.23 | 31.78 | 6.04 | 308.15 | 7.24 | 2.00 | 634.38 | 72.03 | 95.21 | 4.96 | 3.38 | 29.90 | 10.54 | 2.06 |
| *Planigale maculata* | 1230459.35 | 55.55 | 11.30 | 328.66 | 7.65 | 2.00 | 763.54 | 44.76 | 71.04 | 7.59 | 4.07 | 28.57 | 10.58 | 3.62 |
| *Pseudantechinus bilarni* | 134076.80 | 100.00 | 23.05 | 383.31 | 4.75 | 1.00 | 666.63 | 0.00 | 21.05 | 11.08 | 4.09 | 28.52 | 6.08 | 2.60 |
| *Pseudantechinus mimulus* | 3038.45 | 15.68 | 39.99 | 394.36 | 5.94 | 1.00 | 837.20 | 84.32 | 100.00 | 3.33 | 1.88 | 29.05 | 7.54 | 1.34 |
| *Pseudantechinus ningbing* | 207642.66 | 99.99 | 20.74 | 394.48 | 4.00 | 1.05 | 518.37 | 0.00 | 64.88 | 7.89 | 4.13 | 29.18 | 8.36 | 2.25 |
| *Pseudocheirus peregrinus* | 913593.67 | 9.93 | 859.30 | 365.00 | 1.95 | 1.50 | 1217.09 | 81.47 | 55.34 | 6.68 | 4.91 | 26.82 | 27.55 | 5.65 |
| *Pseudochirulus cinereus* | 895.82 | 26.41 | 977.48 | 659.64 | 2.00 | 1.00 | 2011.46 | 0.00 | 100.00 | 2.83 | 1.72 | 23.29 | 26.60 | 2.30 |
| *Pseudochirulus herbertensis* | 3432.75 | 10.51 | 1098.34 | 486.66 | 1.95 | 1.53 | 1385.58 | 0.00 | 100.00 | 4.43 | 3.59 | 23.71 | 64.81 | 3.49 |
| *Pseudomys calabyi* | 20210.66 | 100.00 | 12.00 | 151.95 | 3.25 | 3.04 | 706.25 | 0.00 | 0.00 | 13.58 | 3.21 | 28.10 | 6.27 | 3.31 |
| *Pseudomys delicatulus* | 1805232.47 | 62.00 | 9.78 | 115.69 | 3.10 | 3.64 | 525.11 | 29.92 | 63.89 | 7.31 | 4.42 | 28.74 | 10.38 | 3.18 |
| *Pseudomys desertor* | 5154946.25 | 8.10 | 31.05 | 107.10 | 3.00 | 3.91 | 517.74 | 87.33 | 41.48 | 4.04 | 3.15 | 29.69 | 12.05 | 2.19 |
| *Pseudomys gracilicaudatus* | 337144.31 | 14.58 | 79.53 | 148.60 | 2.96 | 2.94 | 596.93 | 92.06 | 99.54 | 2.31 | 1.68 | 26.34 | 29.35 | 5.08 |
| *Pseudomys johnsoni* | 765965.61 | 58.69 | 12.00 | 142.75 | 3.36 | 3.09 | 686.37 | 40.99 | 91.42 | 5.86 | 3.83 | 29.60 | 8.53 | 2.12 |
| *Pseudomys nanus* | 1004204.99 | 85.51 | 52.19 | 125.70 | 3.00 | 3.13 | 566.25 | 10.91 | 59.44 | 7.38 | 4.16 | 29.29 | 7.66 | 2.61 |
| *Pseudomys patrius* | 404218.19 | 18.37 | 11.00 | 129.55 | 3.35 | 3.19 | 566.48 | 89.82 | 100.00 | 1.99 | 1.50 | 26.38 | 31.84 | 4.34 |
| *Pteropus alecto* | 1197646.69 | 69.31 | 641.12 | 751.93 | 1.10 | 1.00 | 2592.52 | 21.23 | 49.63 | 8.11 | 4.57 | 28.60 | 10.09 | 3.79 |
| *Pteropus conspicillatus* | 29261.81 | 46.29 | 760.71 | 811.93 | 1.00 | 1.43 | 2739.65 | 0.00 | 67.98 | 3.08 | 2.75 | 26.23 | 49.89 | 5.31 |
| *Pteropus scapulatus* | 3028640.67 | 42.45 | 362.00 | 735.85 | 1.00 | 1.55 | 2274.80 | 54.92 | 65.96 | 6.88 | 4.26 | 28.88 | 10.52 | 3.12 |
| *Rattus colletti* | 84775.84 | 99.99 | 151.99 | 82.77 | 6.00 | 4.00 | 511.21 | 0.00 | 0.00 | 9.81 | 4.48 | 28.35 | 6.81 | 4.82 |
| *Rattus leucopus* | 52906.07 | 70.83 | 162.45 | 141.33 | 3.48 | 3.51 | 566.06 | 0.00 | 35.00 | 6.51 | 5.24 | 26.87 | 26.94 | 6.30 |
| *Rattus sordidus* | 208822.08 | 75.84 | 157.50 | 77.00 | 6.00 | 4.00 | 518.08 | 16.14 | 56.95 | 8.04 | 5.86 | 27.06 | 18.79 | 4.56 |
| *Rattus tunneyi* | 759269.65 | 59.38 | 141.40 | 56.00 | 5.55 | 3.81 | 519.11 | 40.10 | 58.13 | 9.12 | 4.33 | 28.54 | 9.66 | 3.65 |
| *Rattus villosissimus* | 2155192.84 | 28.81 | 193.50 | 84.90 | 6.00 | 4.00 | 517.88 | 73.37 | 78.17 | 5.66 | 3.71 | 29.60 | 8.76 | 2.44 |
| *Rhinolophus megaphyllus* | 687704.44 | 28.90 | 8.65 | 855.64 | 1.00 | 1.00 | 2306.44 | 73.79 | 72.94 | 4.98 | 3.66 | 26.79 | 23.75 | 4.57 |
| *Rhinolophus philippinensis* | 104888.89 | 88.75 | 10.91 | 731.80 | 1.03 | 1.01 | 2245.52 | 0.00 | 50.29 | 6.28 | 4.70 | 26.82 | 23.26 | 5.48 |
| *Rhinonicteris aurantia* | 745201.42 | 81.86 | 8.30 | 365.00 | 1.00 | 1.00 | 1853.78 | 1.49 | 40.90 | 8.24 | 4.20 | 29.02 | 7.27 | 2.85 |
| *Saccolaimus flaviventris* | 6045806.61 | 22.07 | 45.25 | 409.59 | 1.01 | 2.00 | 1726.79 | 73.89 | 52.37 | 6.75 | 4.21 | 28.94 | 10.53 | 3.05 |
| *Saccolaimus mixtus* | 36479.89 | 100.00 | 38.29 | 425.00 | 1.01 | 1.28 | 1775.90 | 0.00 | 0.00 | 9.97 | 7.49 | 27.10 | 17.82 | 6.22 |
| *Saccolaimus saccolaimus* | 59827.04 | 71.69 | 43.00 | 417.50 | 1.00 | 1.35 | 1766.34 | 7.09 | 47.76 | 6.27 | 3.36 | 27.28 | 26.53 | 5.62 |
| *Scoteanax rueppellii* | 300801.77 | 10.63 | 26.41 | 677.11 | 1.00 | 0.99 | 2347.61 | 86.59 | 90.38 | 2.76 | 1.98 | 25.85 | 38.00 | 5.75 |
| *Scotorepens balstoni* | 4978418.58 | 3.27 | 11.70 | 412.87 | 1.50 | 1.06 | 1598.90 | 97.24 | 40.23 | 2.55 | 2.37 | 28.83 | 16.87 | 3.02 |
| *Scotorepens greyii* | 4061761.32 | 26.76 | 10.00 | 441.79 | 1.47 | 1.01 | 1958.20 | 67.99 | 68.65 | 6.51 | 3.79 | 29.24 | 9.60 | 2.79 |
| *Scotorepens orion* | 264521.92 | 10.44 | 11.77 | 421.84 | 1.50 | 1.07 | 1655.88 | 84.02 | 60.64 | 2.59 | 2.02 | 25.25 | 31.62 | 4.91 |
| *Scotorepens sanborni* | 585004.30 | 71.07 | 9.92 | 405.94 | 1.50 | 1.06 | 1592.14 | 37.58 | 77.25 | 6.26 | 4.72 | 27.88 | 16.08 | 4.23 |
| *Sminthopsis bindi* | 45582.04 | 100.00 | 17.50 | 344.16 | 6.93 | 1.43 | 579.19 | 0.00 | 0.00 | 12.56 | 3.75 | 28.38 | 6.45 | 3.26 |
| *Sminthopsis leucopus* | 96366.41 | 2.78 | 23.36 | 377.37 | 8.00 | 1.00 | 542.60 | 43.36 | 7.19 | 4.11 | 3.27 | 24.74 | 55.00 | 3.65 |
| *Sminthopsis macroura* | 4299269.39 | 12.33 | 21.30 | 157.66 | 7.00 | 2.00 | 637.48 | 82.20 | 51.06 | 4.39 | 3.18 | 29.62 | 11.35 | 2.02 |
| *Sminthopsis murina* | 1209000.95 | 6.09 | 16.35 | 194.01 | 8.00 | 2.00 | 356.70 | 94.41 | 65.21 | 2.77 | 2.12 | 26.22 | 31.50 | 5.07 |
| *Sminthopsis virginiae* | 270244.43 | 97.07 | 34.00 | 245.97 | 3.31 | 2.22 | 570.07 | 0.24 | 6.70 | 9.88 | 4.99 | 27.93 | 11.34 | 4.20 |
| *Sminthopsis youngsoni* | 1373051.61 | 4.48 | 10.00 | 353.21 | 5.50 | 1.10 | 634.63 | 83.85 | 42.15 | 3.62 | 2.81 | 30.72 | 9.31 | 2.14 |
| *Spilocuscus maculatus* | 47294.73 | 100.00 | 4155.23 | 234.76 | 1.63 | 1.10 | 1656.11 | 0.00 | 0.00 | 8.97 | 6.51 | 27.02 | 20.57 | 5.92 |
| *Syconycteris australis* | 180420.97 | 54.33 | 17.55 | 322.66 | 1.00 | 2.00 | 1191.39 | 37.66 | 70.08 | 5.98 | 4.50 | 26.74 | 26.92 | 5.24 |
| *Tachyglossus aculeatus* | 7656837.58 | 17.35 | 3169.50 | 576.71 | 1.00 | 0.58 | 5687.02 | 78.45 | 41.35 | 6.74 | 4.20 | 28.94 | 10.52 | 3.05 |
| *Taphozous australis* | 36865.20 | 38.63 | 24.22 | 445.94 | 1.00 | 1.00 | 1390.14 | 33.60 | 63.40 | 2.88 | 2.48 | 27.35 | 38.03 | 8.50 |
| *Taphozous georgianus* | 1195675.26 | 68.19 | 24.43 | 365.00 | 1.00 | 1.00 | 788.40 | 13.84 | 38.94 | 7.46 | 4.12 | 29.27 | 7.56 | 2.69 |
| *Taphozous kapalgensis* | 96752.87 | 99.99 | 26.45 | 422.34 | 1.02 | 1.11 | 1648.00 | 0.00 | 18.16 | 11.05 | 3.52 | 28.67 | 5.45 | 4.13 |
| *Taphozous troughtoni* | 741326.76 | 31.93 | 27.48 | 377.33 | 1.00 | 1.00 | 995.69 | 80.33 | 100.00 | 3.33 | 2.93 | 27.99 | 19.71 | 3.55 |
| *Thylogale stigmatica* | 200984.54 | 23.82 | 4305.74 | 415.63 | 1.00 | 1.55 | 1338.66 | 68.06 | 88.40 | 3.96 | 3.17 | 26.06 | 34.12 | 5.41 |
| *Trichosurus vulpecula* | 2696527.42 | 29.71 | 2001.17 | 547.50 | 1.01 | 1.60 | 2008.23 | 69.17 | 56.07 | 8.26 | 4.71 | 28.35 | 11.59 | 3.67 |
| *Uromys caudimaculatus* | 90789.92 | 78.24 | 644.42 | 222.68 | 1.76 | 2.06 | 814.70 | 2.16 | 35.31 | 7.76 | 5.80 | 26.91 | 25.36 | 5.76 |
| *Uromys hadrourus* | 635.19 | 21.39 | 149.11 | 187.02 | 3.00 | 2.67 | 912.50 | 0.00 | 100.00 | 2.92 | 1.85 | 23.74 | 32.09 | 4.30 |
| *Vespadelus caurinus* | 604390.49 | 100.00 | 6.79 | 353.88 | 1.29 | 1.11 | 1435.68 | 0.12 | 43.23 | 8.18 | 4.13 | 29.14 | 7.21 | 2.99 |
| *Vespadelus douglasorum* | 131986.35 | 100.00 | 4.99 | 375.81 | 1.41 | 1.03 | 1591.27 | 0.00 | 12.15 | 8.29 | 4.82 | 29.32 | 8.72 | 3.06 |
| *Vespadelus finlaysoni* | 3433044.36 | 15.34 | 5.00 | 376.92 | 1.32 | 1.05 | 1514.12 | 77.55 | 38.00 | 6.82 | 3.71 | 29.17 | 8.66 | 3.26 |
| *Vespadelus pumilus* | 148337.70 | 17.65 | 5.40 | 397.21 | 1.21 | 1.00 | 1696.64 | 81.12 | 98.92 | 2.68 | 2.04 | 25.43 | 27.64 | 4.61 |
| *Vespadelus troughtoni* | 894611.92 | 28.48 | 5.50 | 367.70 | 1.42 | 1.05 | 1466.86 | 75.23 | 94.38 | 5.75 | 4.54 | 27.31 | 20.15 | 3.73 |
| *Wallabia bicolor* | 1211395.16 | 11.45 | 14812.48 | 273.75 | 1.02 | 1.40 | 1877.38 | 88.32 | 67.66 | 4.95 | 3.80 | 26.91 | 23.80 | 4.76 |
| *Wyulda squamicaudata* | 82065.01 | 100.00 | 1799.21 | 752.44 | 1.01 | 1.00 | 2062.31 | 0.00 | 20.03 | 9.62 | 5.14 | 28.59 | 9.37 | 2.26 |
| *Zyzomys argurus* | 1185306.39 | 62.64 | 45.21 | 182.78 | 2.63 | 1.42 | 704.17 | 25.75 | 52.66 | 7.48 | 3.94 | 28.72 | 10.33 | 3.13 |
| *Zyzomys woodwardi* | 34826.14 | 100.00 | 112.51 | 206.91 | 2.23 | 1.07 | 782.49 | 0.00 | 4.81 | 9.00 | 4.98 | 28.49 | 11.36 | 3.38 |

^*^ Statistically significant (p < 0.05) covariates in the minimum adequate model.

^†^ Covariates in the minimum adequate model.

^a^ BIO8 – WorldClim variable Mean Temperature of Wettest Quarter (Fick & Hijmans, 2017).

^b^ BIO17 – WorldClim variable Precipitation of Driest Quarter (Fick & Hijmans, 2017).

^c^ HII – Human Influence Index; numeric scale from 0–61 (Sanderson et al., 2002).

**APPENDIX S5**

International Union for Conservation of Nature (IUCN) Red List category (IUCN, 2024), population trend, ordinal threat status (10-point numeric scale), and average fitted and latent extinction risk values from 100 model iterations for 128 mammal species native to the Australian Monsoonal Tropics. Latent risk is the difference between the fitted risk value and the current ordinal threat value. Species are listed alphabetically by genus and species. Extinct species were excluded due to insufficient biological or geographic data.

| Scientific Name | IUCN Red List threat category | IUCN Red List population trend | Ordinal threat value | Mean fitted risk | Mean latent risk |
| --- | --- | --- | --- | --- | --- |
| *Acrobates pygmaeus* | Least Concern | Stable | 1 | 0.91 | –0.09 |
| *Aepyprymnus rufescens* | Least Concern | Unknown | 1 | 2.96 | 1.96 |
| *Antechinus adustus* | Least Concern | Decreasing | 2 | 1.26 | –0.74 |
| *Antechinus bellus* | Vulnerable | Decreasing | 6 | 5.37 | –0.63 |
| *Antechinus leo* | Least Concern | Stable | 1 | 3.37 | 2.37 |
| *Cercartetus caudatus* | Least Concern | Stable | 1 | 1.69 | 0.69 |
| *Chaerephon jobensis* | Least Concern | Stable | 1 | 1.52 | 0.52 |
| *Chalinolobus gouldii* | Least Concern | Stable | 1 | 1.12 | 0.12 |
| *Chalinolobus nigrogriseus* | Least Concern | Stable | 1 | 1.06 | 0.06 |
| *Conilurus penicillatus* | Vulnerable | Decreasing | 6 | 2.88 | –3.12 |
| *Dactylopsila trivirgata* | Least Concern | Decreasing | 2 | 2.25 | 0.25 |
| *Dasyurus hallucatus* | Endangered | Decreasing | 8 | 3.70 | –4.30 |
| *Dendrolagus bennettianus* | Near Threatened | Stable | 3 | 2.88 | –0.12 |
| *Dendrolagus lumholtzi* | Near Threatened | Unknown | 3 | 2.74 | –0.26 |
| *Echymipera rufescens* | Least Concern | Decreasing | 2 | 2.57 | 0.57 |
| *Hipposideros ater* | Least Concern | Unknown | 1 | 1.21 | 0.21 |
| *Hipposideros cervinus* | Least Concern | Decreasing | 2 | 1.86 | –0.14 |
| *Hipposideros diadema* | Least Concern | Decreasing | 2 | 1.69 | –0.31 |
| *Hipposideros semoni* | Least Concern | Unknown | 1 | 2.26 | 1.26 |
| *Hipposideros stenotis* | Vulnerable | Unknown | 5 | 2.47 | –2.53 |
| *Hydromys chrysogaster* | Least Concern | Unknown | 1 | 1.57 | 0.57 |
| *Hypsiprymnodon moschatus* | Least Concern | Stable | 1 | 2.00 | 1.00 |
| *Isoodon macrourus* | Least Concern | Stable | 1 | 2.25 | 1.25 |
| *Isoodon obesulus* | Least Concern | Decreasing | 2 | 2.60 | 0.60 |
| *Lagorchestes conspicillatus* | Least Concern | Decreasing | 2 | 2.44 | 0.44 |
| *Leggadina forresti* | Least Concern | Unknown | 1 | 1.33 | 0.33 |
| *Leggadina lakedownensis* | Least Concern | Decreasing | 2 | 1.06 | –0.94 |
| *Macroderma gigas* | Vulnerable | Decreasing | 6 | 2.48 | –3.52 |
| *Macroglossus minimus* | Least Concern | Stable | 1 | 1.15 | 0.15 |
| *Macropus giganteus* | Least Concern | Stable | 1 | 1.97 | 0.97 |
| *Macrotis lagotis* | Vulnerable | Decreasing | 6 | 2.40 | –3.60 |
| *Melomys burtoni* | Least Concern | Stable | 1 | 2.32 | 1.32 |
| *Melomys capensis* | Least Concern | Stable | 1 | 1.20 | 0.20 |
| *Melomys cervinipes* | Least Concern | Stable | 1 | 1.53 | 0.53 |
| *Mesembriomys gouldii* | Vulnerable | Decreasing | 6 | 2.00 | –4.00 |
| *Mesembriomys macrurus* | Near Threatened | Decreasing | 4 | 3.10 | –0.90 |
| *Miniopterus australis* | Least Concern | Stable | 1 | 1.56 | 0.56 |
| *Murina florium* | Least Concern | Unknown | 1 | 1.12 | 0.12 |
| *Myotis macropus* | Least Concern | Unknown | 1 | 1.94 | 0.94 |
| *Notoryctes caurinus* | Least Concern | Stable | 1 | 2.75 | 1.75 |
| *Nyctimene robinsoni* | Least Concern | Unknown | 1 | 2.42 | 1.42 |
| *Nyctophilus arnhemensis* | Least Concern | Stable | 1 | 0.62 | –0.38 |
| *Nyctophilus bifax* | Least Concern | Decreasing | 2 | 1.22 | –0.78 |
| *Nyctophilus geoffroyi* | Least Concern | Stable | 1 | 0.87 | –0.13 |
| *Nyctophilus gouldi* | Least Concern | Decreasing | 2 | 1.74 | –0.26 |
| *Nyctophilus walkeri* | Least Concern | Stable | 1 | 1.48 | 0.48 |
| *Onychogalea unguifera* | Least Concern | Unknown | 1 | 2.25 | 1.25 |
| *Ornithorhynchus anatinus* | Near Threatened | Decreasing | 4 | 3.23 | –0.77 |
| *Perameles nasuta* | Least Concern | Unknown | 1 | 2.28 | 1.28 |
| *Petauroides volans* | Vulnerable | Decreasing | 6 | 3.45 | –2.55 |
| *Petaurus breviceps* | Least Concern | Stable | 1 | 2.43 | 1.43 |
| *Petaurus norfolcensis* | Least Concern | Decreasing | 2 | 2.41 | 0.41 |
| *Petrogale assimilis* | Least Concern | Stable | 1 | 2.97 | 1.97 |
| *Petrogale brachyotis* | Least Concern | Unknown | 1 | 3.38 | 2.38 |
| *Petrogale burbidgei* | Near Threatened | Unknown | 3 | 4.25 | 1.25 |
| *Petrogale concinna* | Endangered | Decreasing | 8 | 3.93 | –4.07 |
| *Petrogale godmani* | Near Threatened | Decreasing | 4 | 2.32 | –1.68 |
| *Petrogale mareeba* | Near Threatened | Stable | 3 | 2.65 | –0.35 |
| *Petrogale purpureicollis* | Near Threatened | Decreasing | 4 | 2.97 | –1.03 |
| *Petrogale sharmani* | Vulnerable | Unknown | 5 | 3.32 | –1.68 |
| *Petropseudes dahli* | Least Concern | Unknown | 1 | 1.68 | 0.68 |
| *Phalanger mimicus* | Least Concern | Decreasing | 2 | 3.72 | 1.72 |
| *Phascogale pirata* | Vulnerable | Unknown | 5 | 4.82 | –0.18 |
| *Phascogale tapoatafa* | Near Threatened | Decreasing | 4 | 2.83 | –1.17 |
| *Phascolarctos cinereus* | Vulnerable | Decreasing | 6 | 3.07 | –2.93 |
| *Phoniscus papuensis* | Vulnerable | Unknown | 5 | 1.02 | –3.98 |
| *Pipistrellus adamsi* | Least Concern | Stable | 1 | 0.62 | –0.38 |
| *Pipistrellus westralis* | Least Concern | Stable | 1 | 0.18 | –0.82 |
| *Planigale ingrami* | Least Concern | Stable | 1 | 1.26 | 0.26 |
| *Planigale maculata* | Least Concern | Stable | 1 | 1.88 | 0.88 |
| *Pseudantechinus bilarni* | Least Concern | Decreasing | 2 | 2.88 | 0.88 |
| *Pseudantechinus mimulus* | Near Threatened | Decreasing | 4 | 3.53 | –0.47 |
| *Pseudantechinus ningbing* | Least Concern | Unknown | 1 | 1.52 | 0.52 |
| *Pseudocheirus peregrinus* | Least Concern | Stable | 1 | 2.24 | 1.24 |
| *Pseudochirulus cinereus* | Near Threatened | Decreasing | 4 | 3.79 | –0.21 |
| *Pseudochirulus herbertensis* | Least Concern | Stable | 1 | 2.73 | 1.73 |
| *Pseudomys calabyi* | Vulnerable | Decreasing | 6 | 4.99 | –1.01 |
| *Pseudomys delicatulus* | Least Concern | Unknown | 1 | 0.66 | –0.34 |
| *Pseudomys desertor* | Least Concern | Decreasing | 2 | 0.77 | –1.23 |
| *Pseudomys gracilicaudatus* | Least Concern | Decreasing | 2 | 2.10 | 0.10 |
| *Pseudomys johnsoni* | Least Concern | Stable | 1 | 0.93 | –0.07 |
| *Pseudomys nanus* | Least Concern | Decreasing | 2 | 1.51 | –0.49 |
| *Pseudomys patrius* | Least Concern | Unknown | 1 | 1.01 | 0.01 |
| *Pteropus alecto* | Least Concern | Unknown | 1 | 3.04 | 2.04 |
| *Pteropus conspicillatus* | Endangered | Decreasing | 8 | 3.11 | –4.89 |
| *Pteropus scapulatus* | Least Concern | Unknown | 1 | 2.89 | 1.89 |
| *Rattus colletti* | Least Concern | Unknown | 1 | 2.76 | 1.76 |
| *Rattus leucopus* | Least Concern | Stable | 1 | 1.25 | 0.25 |
| *Rattus sordidus* | Least Concern | Stable | 1 | 1.13 | 0.13 |
| *Rattus tunneyi* | Least Concern | Unknown | 1 | 1.80 | 0.80 |
| *Rattus villosissimus* | Least Concern | Unknown | 1 | 1.30 | 0.30 |
| *Rhinolophus megaphyllus* | Least Concern | Unknown | 1 | 1.91 | 0.91 |
| *Rhinolophus philippinensis* | Least Concern | Unknown | 1 | 1.22 | 0.22 |
| *Rhinonicteris aurantia* | Least Concern | Unknown | 1 | 1.27 | 0.27 |
| *Saccolaimus flaviventris* | Least Concern | Decreasing | 2 | 1.92 | –0.08 |
| *Saccolaimus mixtus* | Near Threatened | Decreasing | 4 | 1.69 | –2.31 |
| *Saccolaimus saccolaimus* | Least Concern | Stable | 1 | 3.09 | 2.09 |
| *Scoteanax rueppellii* | Least Concern | Stable | 1 | 2.68 | 1.68 |
| *Scotorepens balstoni* | Least Concern | Decreasing | 2 | 1.01 | –0.99 |
| *Scotorepens greyii* | Least Concern | Stable | 1 | 1.14 | 0.14 |
| *Scotorepens orion* | Least Concern | Unknown | 1 | 1.96 | 0.96 |
| *Scotorepens sanborni* | Least Concern | Stable | 1 | 0.86 | –0.14 |
| *Sminthopsis bindi* | Near Threatened | Decreasing | 4 | 4.49 | 0.49 |
| *Sminthopsis leucopus* | Least Concern | Stable | 1 | 2.09 | 1.09 |
| *Sminthopsis macroura* | Least Concern | Unknown | 1 | 0.63 | –0.37 |
| *Sminthopsis murina* | Least Concern | Decreasing | 2 | 1.37 | –0.63 |
| *Sminthopsis virginiae* | Least Concern | Stable | 1 | 2.35 | 1.35 |
| *Sminthopsis youngsoni* | Least Concern | Stable | 1 | 1.09 | 0.09 |
| *Spilocuscus maculatus* | Least Concern | Stable | 1 | 1.61 | 0.61 |
| *Syconycteris australis* | Least Concern | Stable | 1 | 1.79 | 0.79 |
| *Tachyglossus aculeatus* | Least Concern | Stable | 1 | 1.77 | 0.77 |
| *Taphozous australis* | Near Threatened | Decreasing | 4 | 2.43 | –1.57 |
| *Taphozous georgianus* | Least Concern | Stable | 1 | 1.60 | 0.60 |
| *Taphozous kapalgensis* | Least Concern | Unknown | 1 | 3.59 | 2.59 |
| *Taphozous troughtoni* | Least Concern | Decreasing | 2 | 0.91 | –1.09 |
| *Thylogale stigmatica* | Least Concern | Decreasing | 2 | 2.79 | 0.79 |
| *Trichosurus vulpecula* | Least Concern | Decreasing | 2 | 2.98 | 0.98 |
| *Uromys caudimaculatus* | Least Concern | Stable | 1 | 1.97 | 0.97 |
| *Uromys hadrourus* | Near Threatened | Stable | 3 | 3.04 | 0.04 |
| *Vespadelus caurinus* | Least Concern | Stable | 1 | 1.04 | 0.04 |
| *Vespadelus douglasorum* | Least Concern | Unknown | 1 | 0.82 | –0.18 |
| *Vespadelus finlaysoni* | Least Concern | Stable | 1 | 1.01 | 0.01 |
| *Vespadelus pumilus* | Least Concern | Stable | 1 | 1.41 | 0.41 |
| *Vespadelus troughtoni* | Least Concern | Stable | 1 | 0.35 | –0.65 |
| *Wallabia bicolor* | Least Concern | Increasing | 1 | 1.63 | 0.63 |
| *Wyulda squamicaudata* | Near Threatened | Decreasing | 4 | 3.31 | –0.69 |
| *Zyzomys argurus* | Least Concern | Stable | 1 | 1.51 | 0.51 |
| *Zyzomys woodwardi* | Least Concern | Stable | 1 | 1.97 | 0.97 |

**APPENDIX S6**

Representation targets assigned to mammal species native to the Australian Monsoonal Tropics (AMT) when prioritising current extinction risk and positive latent extinction risk. For the current risk criterion, ‘Threat value’ is based on a 10-point numeric scale combining species’ threat category and population trend from the International Union for Conservation of Nature (IUCN) Red List (IUCN, 2024). For the positive latent risk criterion, ‘Threat value’ is the difference between the fitted risk value and current ordinal threat value as per the numeric scale. ‘Representation target’ is the percentage of species’ geographic range in the AMT to be secured under each prioritization scenario (i.e., combination of objective and criterion), calculated as the weighted sum of the target species’ threat value and the proportion of the species’ geographic distribution in the AMT.

| Scientific Name | Criterion | Threat value | % geographic range overlap with AMT | Representation target (%) |
| --- | --- | --- | --- | --- |
| *Petrogale concinna* | Current risk | 8 | 100.00 | 75.00 |
| *Pseudomys calabyi* | Current risk | 6 | 100.00 | 65.00 |
| *Antechinus bellus* | Current risk | 6 | 99.99 | 65.00 |
| *Conilurus penicillatus* | Current risk | 6 | 99.98 | 65.00 |
| *Pteropus conspicillatus* | Current risk | 8 | 46.29 | 63.52 |
| *Dasyurus hallucatus* | Current risk | 8 | 40.44 | 61.18 |
| *Mesembriomys gouldii* | Current risk | 6 | 89.62 | 60.85 |
| *Phascogale pirata* | Current risk | 5 | 99.99 | 55.00 |
| *Hipposideros stenotis* | Current risk | 5 | 97.32 | 53.93 |
| *Macroderma gigas* | Current risk | 6 | 64.46 | 50.79 |
| *Petrogale sharmani* | Current risk | 5 | 78.31 | 46.32 |
| *Macrotis lagotis* | Current risk | 6 | 12.06 | 29.83 |
| *Phascolarctos cinereus* | Current risk | 6 | 8.85 | 28.54 |
| *Petauroides volans* | Current risk | 6 | 7.97 | 28.19 |
| *Phoniscus papuensis* | Current risk | 5 | 26.85 | 25.74 |
|  |  |  |  |  |
| *Antechinus leo* | Positive latent risk | 2.37 | 99.99 | 75.00 |
| *Petrogale brachyotis* | Positive latent risk | 2.38 | 99.99 | 75.00 |
| *Taphozous kapalgensis* | Positive latent risk | 2.59 | 99.99 | 75.00 |
| *Rattus colletti* | Positive latent risk | 1.76 | 99.99 | 57.51 |
| *Saccolaimus saccolaimus* | Positive latent risk | 2.09 | 71.69 | 57.14 |
| *Phalanger mimicus* | Positive latent risk | 1.72 | 99.99 | 56.04 |
| *Pteropus alecto* | Positive latent risk | 2.04 | 69.31 | 54.39 |
| *Petrogale assimilis* | Positive latent risk | 1.97 | 47.06 | 43.26 |
| *Pteropus scapulatus* | Positive latent risk | 1.89 | 42.45 | 38.95 |
| *Aepyprymnus rufescens* | Positive latent risk | 1.96 | 15.70 | 30.43 |
| *Notoryctes caurinus* | Positive latent risk | 1.75 | 17.44 | 24.10 |
| *Pseudochirulus herbertensis* | Positive latent risk | 1.73 | 10.51 | 20.59 |
| *Scoteanax rueppellii* | Positive latent risk | 1.68 | 10.63 | 19.25 |

**APPENDIX S7**

Map depicting mammal species richness in the Australian Monsoonal Tropics (AMT), calculated for 128 mammal species native to the AMT and included in the comparative extinction risk models and systematic conservation planning components of this study.


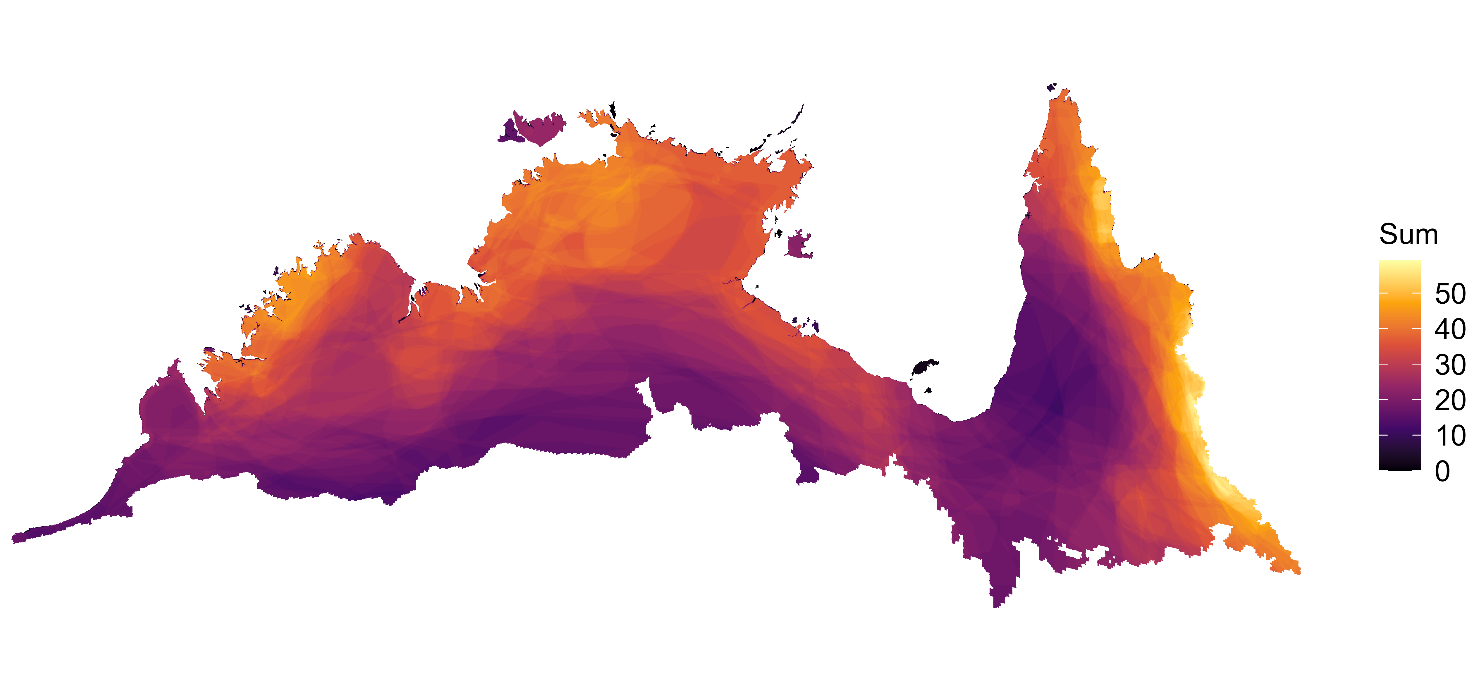


**REFERENCES**

ABS. (2018). *Regional statistics by LGA: median employee income 2011-2018*. Australian Bureau of Statistics. <https://api.data.abs.gov.au/data/ABS_REGIONAL_LGA2018/ERP_19.LGA2018..A/>

Brundrett, M. C. (2017). Global diversity and importance of mycorrhizal and nonmycorrhizal plants. *Biogeography of mycorrhizal symbiosis*, 533-556.

DCCEEW. (2021). *Interim Biogeographic Regionalisation for Australia version 7.0*. Department of Climate Change, Energy, the Environment, and Water. Australian Government. <https://www.dcceew.gov.au/environment/land/nrs/science/ibra>

Fick, S. E., & Hijmans, R. J. (2017). WorldClim 2: New 1‐km spatial resolution climate surfaces for global land areas. *International Journal of Climatology*, *37*(12), 4302-4315.

IUCN. (2024). *The IUCN Red List of Threatened Species. Version 2024-1*. Retrieved 20 May 2024 from <https://www.iucnredlist.org>

Sanderson, E. W., Jaiteh, M., Levy, M. A., Redford, K. H., Wannebo, A. V., & Woolmer, G. (2002). The Human Footprint and the Last of the Wild: The human footprint is a global map of human influence on the land surface, which suggests that human beings are stewards of nature, whether we like it or not. *BioScience*, *52*(10), 891-904.
